# Selective Targeting of Protein Kinase C (PKC)-θ Nuclear Translocation Reduces Mesenchymal Gene Signatures and Reinvigorates Dysfunctional CD8^+^ T Cells in Immunotherapy-Resistant and Metastatic Cancers

**DOI:** 10.3390/cancers14061596

**Published:** 2022-03-21

**Authors:** Jenny Dunn, Robert D. McCuaig, Abel H. Y. Tan, Wen Juan Tu, Fan Wu, Kylie M. Wagstaff, Anjum Zafar, Sayed Ali, Himanshu Diwakar, Jane E. Dahlstrom, Elaine G. Bean, Jade K. Forwood, Sofiya Tsimbalyuk, Emily M. Cross, Kristine Hardy, Amanda L. Bain, Elizabeth Ahern, Riccardo Dolcetti, Roberta Mazzieri, Desmond Yip, Melissa Eastgate, Laeeq Malik, Peter Milburn, David A. Jans, Sudha Rao

**Affiliations:** 1 Gene Regulation and Translational Medicine Laboratory, QIMR Berghofer Medical Research Institute, Herston, QLD 4006, Australia; jenny.dunn@qimrberghofer.edu.au (J.D.); robert.mccuaig@qimrberghofer.edu.au (R.D.M.); wenjuan.tu@qimrberghofer.edu.au (W.J.T.); amanda.bain@qimrberghofer.edu.au (A.L.B.); 2 Melanie Swan Memorial Translational Centre, Faculty of Science and Technology, University of Canberra, Canberra, ACT 2617, Australia; abelthy@hotmail.com (A.H.Y.T.); fan.wu@menarini-cn.com (F.W.); anjum_zfr@yahoo.com (A.Z.); kristine.hardy@anu.edu.au (K.H.); 3 Cancer Targeting and Nuclear Therapeutics Lab, Department of Biochemistry and Molecular Biology, Monash Biomedicine Discovery Institute, Monash University, Melbourne, VIC 3800, Australia; kylie.wagstaff@monash.edu; 4 Medical Oncology, St John of God Midland Public and Private Hospitals, Perth, WA 6056, Australia; sayed.ali@sjog.org.au; 5 Woden Specialist Medical Centre, I-MED Radiology Network, Canberra, ACT 2606, Australia; drhimanshu.diwakar@gmail.com; 6 Anatomical Pathology, ACT Pathology, The Canberra Hospital, Canberra Health Services, Canberra, ACT 2605, Australia; jane.e.dahlstrom@act.gov.au (J.E.D.); elaine.bean@act.gov.au (E.G.B.); 7 ANU Medical School, College of Health and Medicine, The Australian National University, Canberra, ACT 0200, Australia; desmond.yip@act.gov.au (D.Y.); laeeq.malik@act.gov.au (L.M.); 8 The John Curtin School of Medical Research, The Australian National University, Canberra, ACT 0200, Australia; peter.milburn@anu.edu.au; 9 School of Dentistry and Medical Sciences, Charles Sturt University, Wagga Wagga, NSW 2678, Australia; jforwood@csu.edu.au (J.K.F.); stsimbalyuk@csu.edu.au (S.T.); emcross@csu.edu.au (E.M.C.); 10 Department of Medical Oncology, Monash Health and Monash University, Melbourne, VIC 3168, Australia; elizabeth.ahern@monash.edu; 11 Cancer Immunoregulation and Immunotherapy Laboratory, QIMR Berghofer Medical Research Institute, Herston, QLD 4006, Australia; 12 Peter MacCallum Cancer Centre, Melbourne, VIC 3000, Australia; riccardo.dolcetti@petermac.org (R.D.); r.mazzieri@uq.edu.au (R.M.); 13 Sir Peter MacCallum Department of Oncology, The University of Melbourne, Melbourne, VIC 3010, Australia; 14 Department of Microbiology and Immunology, The University of Melbourne, Melbourne, VIC 3010, Australia; 15 The University of Queensland Diamantina Institute, Translational Research Institute, The University of Queensland, Brisbane, QLD 4102, Australia; 16 Department of Medical Oncology, Canberra Health Services, The Canberra Hospital, Canberra, ACT 2605, Australia; 17 Department of Medical Oncology, Royal Brisbane and Women’s Hospital, Brisbane, QLD 4029, Australia; melissa.eastgate@health.qld.gov.au; 18 Faculty of Medicine, University of Queensland, Herston, QLD 4006, Australia; 19 Nuclear Signaling Lab, Department of Biochemistry and Molecular Biology, Monash Biomedicine Discovery Institute, Monash University, Melbourne, VIC 3800, Australia; david.jans@monash.edu

**Keywords:** breast cancer, cancer stem cell, epithelial-to-mesenchymal transition, immunotherapy, melanoma, metastasis, nuclear translocation, protein kinase C (PKC)-θ, T cell, resistance

## Abstract

**Simple Summary:**

Some important signaling proteins that control how cells grow and behave not only act in the cytoplasm but also in the nucleus, where they tether to chromatin. This is especially true for protein kinase C (PKC)-θ, which acts in the nucleus to mediate cancer hallmarks that drive metastasis and in normal T cells. However, current PKC-θ inhibitors are either non-specific or target only its cytoplasmic function. In a bid to develop a novel class of PKC-θ inhibitor that maintains cytoplasmic signaling but inhibits its nuclear function, here we present a novel PKC-θ inhibitor (nPKC-θi2) that specifically inhibits nuclear translocation of PKC-θ without interrupting normal signaling in healthy T cells. We show for the first time that nPKC-θ mediates immunotherapy resistance via its activity in circulating tumor cells and dysfunctional CD8^+^ T cells. Our novel inhibitor provides a means to target this process by simultaneously overcoming T-cell exhaustion and cancer stem cell burden. As part of a sequential approach with other therapies, this work paves the way for improving outcomes in cancer patients with immunotherapy-resistant relapse and metastasis.

**Abstract:**

Protein kinase C (PKC)-θ is a serine/threonine kinase with both cytoplasmic and nuclear functions. Nuclear chromatin-associated PKC-θ (nPKC-θ) is increasingly recognized to be pathogenic in cancer, whereas its cytoplasmic signaling is restricted to normal T-cell function. Here we show that nPKC-θ is enriched in circulating tumor cells (CTCs) in patients with triple-negative breast cancer (TNBC) brain metastases and immunotherapy-resistant metastatic melanoma and is associated with poor survival in immunotherapy-resistant disease. To target nPKC-θ, we designed a novel PKC-θ peptide inhibitor (nPKC-θi2) that selectively inhibits nPKC-θ nuclear translocation but not PKC-θ signaling in healthy T cells. Targeting nPKC-θ reduced mesenchymal cancer stem cell signatures in immunotherapy-resistant CTCs and TNBC xenografts. PKC-θ was also enriched in the nuclei of CD8^+^ T cells isolated from stage IV immunotherapy-resistant metastatic cancer patients. We show for the first time that nPKC-θ complexes with ZEB1, a key repressive transcription factor in epithelial-to-mesenchymal transition (EMT), in immunotherapy-resistant dysfunctional PD1^+^/CD8^+^ T cells. nPKC-θi2 inhibited the ZEB1/PKC-θ repressive complex to induce cytokine production in CD8^+^ T cells isolated from patients with immunotherapy-resistant disease. These data establish for the first time that nPKC-θ mediates immunotherapy resistance via its activity in CTCs and dysfunctional CD8^+^ T cells. Disrupting nPKC-θ but retaining its cytoplasmic function may offer a means to target metastases in combination with chemotherapy or immunotherapy.

## 1. Introduction

Some signaling kinases not only transiently phosphorylate their cytoplasmic substrates but also act as chromatin-associated nuclear kinases. First described for Hog1 [1,2], other kinases including c-Jun N-terminal kinases, protein kinase B and C (PKB/PKC), p38 mitogen-activated protein kinase, and IκB kinase are now known to have dual cytoplasmic and nuclear roles [3]. These kinases form stable complexes with chromatin to directly regulate transcription by recruiting transcription factors that in turn recruit RNA polymerase II machinery. Analogous to these studies, we previously reported that PKC-θ directly tethers to chromatin, where it functions as a structural adaptor or locally phosphorylates histones (e.g., H2BSer32p) or transcription factors (e.g., NF-κB p65) [4,5].

In breast cancer, we showed that PKC-θ tethers to the chromatin of inducible epithelial-to-mesenchymal (EMT) genes to induce cancer stem cell (CSC) formation [6]. Furthermore, nuclear PKC-θ directly phosphorylates lysine-specific demethylase 1 (LSD1) to regulate inducible EMT programs in metastatic breast cancer [7]. While nuclear chromatin-associated (n)PKC-θ is associated with breast cancer and other diseases, in healthy T cells, cytoplasmic PKC-θ selectively translocates to the immunological synapse during T-cell receptor (TCR)/CD28 co-stimulation [8,9,10,11,12], where it transiently phosphorylates and regulates transcription factors (e.g., NF-κB, AP-1, and NFAT) during T-cell activation [10,13,14]. Therefore, nPKC-θ appears to be a feature of the aberrant gene expression programs that drive cancer progression, metastasis, and recurrence, with its cytoplasmic localization restricted to normal T-cell function.

Most existing PKC-θ inhibitors such as sotrasautrin (AEB071), R524, and enzastaurin are competitive ATP inhibitors that block the cytoplasmic, enzymatic action of PKC-θ [15,16,17,18,19]. They are also mostly non-specific; AEB071 targets several PKC isoforms, and R524 and enzastaurin target PKC-θ/PKC-α and PKC-θ/PKC-β, respectively [15,16,17,18,19]. C27 and C20 are competitive ATP inhibitors that specifically target PKC-θ activity [20] and thus also affect normal T-cell processes [20]. Novel classes of PKC-θ inhibitor that maintain cytoplasmic signaling but inhibit its nuclear function are required to limit off-target effects.

While metastasis-initiating cells such as mesenchymal CSCs and circulating tumor cells (CTCs) play critical roles in relapse, metastasis, and resistance to therapy, T-cell dysfunction is often a cancer hallmark and an underlying mechanism of immunotherapy resistance. Dysfunctional T cells are characterized by reduced proliferative capacity, overexpression of checkpoint inhibitory receptors, and reduced production of cytokines such as tumor necrosis factor-α (TNF-α) and interferon-γ (IFN-γ) [21]. While dysfunctional T cells retain residual cytotoxic function, they fail to effectively eliminate primary infections or tumors. Anti-PD-1 immunotherapy partially reinvigorates dysfunctional T cells, though durable invigoration remains problematic and intrinsic and acquired resistance are common [22,23]. The peripheral blood of cancer patients, particularly those with extensive disease, is enriched in CD8^+^ T cells expressing exhaustion and immunotherapy resistance proteins including PD-1, TIM3, TIGIT, and LAG3 [24,25,26]. While the normal function of PKC-θ in T cells is well established, its roles in immunotherapy resistance and dysfunctional T cells have yet to be explored.

Here we show that nPKC-θ is enriched alongside key metastasis proteins in immunotherapy-resistant CTCs isolated from patients with metastases and immunotherapy-resistant metastatic melanoma, where it is associated with poor survival outcomes. Furthermore, nPKC-θ is enriched in CD8^+^ T cells isolated from metastatic cancer patients resistant to immunotherapy treatment, where it is complexed with ZEB1, a key repressive transcription factor in EMT. Based on our previous identification of a nuclear localization signal (NLS) motif in PKC-θ [2], we derive a novel PKC-θ inhibitor (nPKC-θi2) that specifically inhibits nuclear translocation of PKC-θ without interrupting normal catalytic signaling in healthy T cells. Inhibition of nPKC-θ partially overcomes metastatic tumor growth by inhibiting mesenchymal signatures and CSC production in murine triple-negative breast cancers (TNBCs). In addition, nPKC-θi2 inhibits the repressive ZEB1/PKC-θ complex and induces cytokine production in CD8^+^ T cells isolated from patients with immunotherapy-resistant disease. Overall, targeting nuclear kinase pathways such as PKC-θ for anti-tumor therapy could form part of a sequential approach that targets CTCs and reinvigorates dysfunctional T cells in patients with immunotherapy-resistant relapse and metastasis.

## 2. Materials and Methods

### 2.1. Cell Culture

MDA-MB-231 (ATCC HTB-26), MCF-7 (ATCC HTB-22), B16-F10 (ATCC CRL-6475), 4T1 (ATCC CRL-2539), and RPMI-7951 (ATCC HTB-66) cells were cultured in high-glucose DMEM supplemented with 2 mM L-glutamine, 1 × penicillin-streptomycin-neomycin (PSN) (Gibco, Thermo Fisher Scientific, Waltham, MA, USA), and 10% heat-inactivated fetal calf serum (FCS). SK-MEL-3 (ATCC HTB-69) cells were cultured in complete McCoy’s 5a medium supplemented with 2 mM L-glutamine, 1 × PSN, and 15% heat-inactivated FCS. The human Jurkat T-cell line (clone E6-1, ATCC TIB-152) was cultured in complete RPMI medium (Gibco) supplemented with heat-inactivated 10% FCS, 2 mM L-glutamine, 1 × PSN, and 10 mM HEPES (Gibco).

### 2.2. MCF-7 Inducible Model (MCF-7-IM)

Human MCF-7 breast cancer cells were activated with phorbol 12-myristate 13-acetate (PMA; 0.65 ng/mL) (Sigma-Aldrich, St. Louis, MO, USA) or PMA and transforming growth factor β (TGF-β; 20 ng/mL) (R & D Systems, Minneapolis, MN, USA) for 60 h to induce EMT and mesenchymal-like MCF-7 cells. MCF-7 cells were pre-treated with vehicle, nPKC-θi2, or C27 inhibitor as indicated for 24 h before activation.

### 2.3. Reconstitution of Nuclear Import

Nuclear import of fluorescently labelled PKC-θ (DTAF-PKC-θ) was reconstituted in vitro in mechanically perforated HTC cells in the presence (+) or absence (−) of exogenous cytosol and an ATP regeneration system as previously shown [27,28]. Confocal laser scanning microscopy (CLSM) images were acquired periodically for measurement of accumulation of DTAF-PKC-θ into intact nuclei. Nuclear integrity was confirmed by the exclusion of Texas red-labelled 70 kDa dextran (TR70).

### 2.4. AlphaScreen^®^ Binding Assay

An AlphaScreen assay was performed in triplicate as previously described to quantify interactions between PKC-θ and importin proteins [29]. About 30 nM of His_6_-PKC-θ and increasing concentrations of the indicated biotinylated GST-Imp(ortin)s were used. All additions and incubations were performed under subdued lighting. The assay was measured on a PerkinElmer EnSpire plate reader (PerkinElmer, Waltham, MA, USA), triplicate values averaged, and three parameter sigmoidal curves fitted using GraphPad Prism (GraphPad Software, La Jolla, CA, USA). As previously, quenched values in the “hooking zone” were excluded from the analysis [29,30,31].

### 2.5. CD8^+^ and CTC Enrichment from Blood

CD8^+^ T cells and CTCs were isolated from blood using the RosetteSep™ human CD8^+^ enrichment (15063, Stemcell Technologies, Vancouver, BC, Canada) or CD45^+^ depletion cocktail, Lymphoprep™, and SepMate™ tubes (Stemcell Technologies) according to the manufacturer’s protocol. Briefly, RosetteSep™ cocktail antibodies were incubated with 30–40 mL of blood. Samples were then layered on Lymphoprep™ solution in SepMate™ tubes. The monolayer was collected and red blood cells lysed with red blood cell lysis buffer (Millipore, Sigma-Aldrich) for downstream applications. All human experimental procedures were performed in accordance with the guidelines and regulations approved by the ACT Health Research Ethics and Governance Office Human Research Ethics Committee (ethics IDs ETH.5.16.073 & ETH.11.15.217; P3634–RBWH: Immunotherapy STARS (stratifying treatment resistance and sensitivity). All patients provided written informed consent prior to inclusion. Melanoma patients were recruited based on immunotherapy response as measured by RECIST v1.1 criteria [32] and classified into responder or resistant groups using the RECIST 1.1 analysis described in [33]. Patient cohorts were further divided into complete response (CR), partial response (PR), stable disease (SD), and progressive disease (PD), where CR, PR, and SD were considered responders and PD resistant. Samples were collected at baseline and then either monthly or three-monthly thereafter where possible. Total of 24 melanoma patients and 10 metastatic breast cancer patients were recruited for liquid biopsy analysis.

### 2.6. Cell and Tissue Processing and Immunofluorescence Microscopy

CTCs from metastatic melanoma patients were pre-enriched using the RosetteSep™ Human CD45 Depletion Kit (15162, Stemcell Technologies) as described above. Enriched cells were then cytospun onto coverslips pre-treated with poly-l-lysine, fixed, and stored in PBS for staining.

Immunofluorescence (IF) imaging and analysis were carried out using previously established and optimized protocols [4,6,7,34,35,36]. Briefly, CTCs were permeabilized by incubating with 0.5% Triton X-100 for 20 min, probed with a cocktail of primary antibodies as indicated, and visualized with donkey anti-rabbit Alexa Fluor (AF) 488, anti-mouse AF 568, and anti-goat AF 647 secondary antibodies or with the OPAL tyramide system (BOND-RX, Leica Microsystems, Wetzlar, Germany). Coverslips were mounted on glass microscope slides with ProLong Diamond Antifade reagent (Life Technologies, Thermo Fisher Scientific). Protein targets were localized by confocal laser scanning microscopy. Single 0.5 μm sections were obtained using a 100× oil immersion lens on a Leica DMI8 microscope running LAX software. The final image was obtained by averaging four sequential images of the same section. Digital images were analyzed using ImageJ software (ImageJ, NIH, Bethesda, MD, USA) to determine either the nuclear fluorescence intensity (NFI), the total cytoplasmic fluorescence intensity (TCFI), or the total fluorescence intensity (TFI). Andor WD Revolution (Oxford Instruments, Abingdon, UK) super-resolution imaging was carried out according to normal specifications for the system with sample preparation identical to that for immunofluorescence.

Formalin-fixed, paraffin-embedded (FFPE) tissue biopsies of primary melanomas were processed using the BOND-RX (Leica Microsystems) for immunofluorescence staining using the instrument protocol: ER2 for 20 min at 100 °C with Epitope Retrieval Solution 2 (pH9 EDTA-based solution). Protein targets were localized by confocal laser scanning microscopy as above.

### 2.7. Applied Spectral Imaging (ASI) Digital Pathology

Using the ASI Digital Pathology system, touching cells were automatically segmented, signal expression quantified, and results per cell and over the entire scanned region displayed. For high-throughput microscopy, protein targets were localized by confocal laser scanning microscopy. Single 0.5 μm sections were obtained using an Olympus ASI automated microscope with either a 20× objective, 60× objective, or a 100× oil immersion objective running ASI software. The final image was obtained by employing a high-throughput automated stage with ASI spectral capture software. Digital images were analyzed using automated ASI software (Applied Spectral Imaging, Carlsbad, CA, USA) to automatically determine the distribution and intensities with automatic thresholding and background correction of either the mean NFI, CFI, or FI as well as the percentage population of cells expressing the analyzed proteins.

### 2.8. Proximity Ligation Assay (PLA)

Using our previously established protocols [34,35], the Duolink proximity ligation assay was employed using PLA probe anti-mouse PLUS (DUO92001), PLA probe anti-rabbit MINUS (DUO92005), and Duolink In Situ Detection Reagent Red Kit (DUO92008) (Sigma-Aldrich). Cells were fixed, permeabilized, and incubated with primary antibodies targeting PKC-θ-Thr538p and ZEB1. Cells were processed according to the manufacturer’s recommendations. Finally, coverslips were mounted onto slides, which were examined as above.

### 2.9. Nuclear to Cytoplasmic Fluorescence Ratio (Fn/c) Analysis

Digital confocal images were analyzed using Fiji-ImageJ software [37] to determine the NFI, TCFI, TFI, or the nuclear to cytoplasmic fluorescence ratio (Fn/c) using the equation: Fn/c = (Fn − Fb)/(Fc − Fb), where Fn is nuclear fluorescence, Fc is cytoplasmic fluorescence, and Fb is background fluorescence. A minimum of *n* = 20 cells were analyzed for each sample set. The Mann–Whitney non-parametric test (GraphPad Prism, GraphPad Software, San Diego, CA) was used to determine significant differences between pairs of datasets and one-way ANOVA for group analysis.

### 2.10. PKC-θ Activity Assay

Recombinant PKC-θ was used in the PKC kinase activity kit (Enzo Life Sciences, Farmingdale, NY, USA) according to the manufacturer’s protocol. Briefly, 5 ng of recombinant PKC-θ was co-incubated with PKC inhibitors in a substrate-coated plate for 15 min at 30 °C before the addition of ATP for the kinase reaction. Subsequently, 1hosphor-substrate antibody, secondary antibody-HRP conjugate, and TMB substrate were added in between washes. Finally, a stop solution was added, and the absorbance value was measured using a spectrophotometer at 450 nm. Relative percentage of PKC activity was calculated using the formula: (absorbance value with inhibitor/absorbance value without inhibitor) × 100%.

### 2.11. WST-1 Cell Viability Assay

Cells were seeded at optimized densities into 96-well flat-bottomed plates in complete medium overnight. After 24 h, the medium was replaced with various concentrations of PKC-θ inhibitors and treated for 72 h. The inhibitors were then removed and WST-1 cell proliferation reagent (Sigma-Aldrich) added 1:10 with complete medium to each well. The absorbance was measured using a spectrophotometer at 450 nm at 1 h intervals for up to 4 h after the addition of WST-1 reagent. GraphPad Prism (GraphPad Software, La Jolla, CA, USA) was used to plot a non-linear regression using the log(inhibitor) vs. normalized response–variable slope function to determine the EC_50_ of the inhibitors.

### 2.12. IncuCyte^®^ Scratch Wound Assay

MDA-MB-231 cells were seeded in low serum (2%) DMEM at a density of 2.5 × 10^4^ cells per well (100 µL/well) in 96-well IncuCyte^®^ ImageLock plates (Sartorius, Göttingen, Germany) and allowed to adhere overnight. The following day, the center of each well was scratched using the WoundMaker Tool (Sartorius) and the wounded monolayer washed with low serum medium to remove non-adherent cells before adding inhibitors (100 µL/well). Wound healing images were acquired by real-time imaging using the IncuCyte Zoom live cell analysis system every 6 h for 24 h. Relative wound density was analyzed using IncuCyte Zoom and graphed using GraphPad Prism.

### 2.13. In Vivo Mouse Xenograft Model

Total of 2 × 10^6^ MDA-MB-231 cells were injected subcutaneously into the mammary glands of five-week-old BALB/c nude mice (Animal Resources Centre, Perth) in 1:1 PBS and BD Matrigel matrix (Corning, Corning, NY, USA). Treatment was started 15 days after cell implantation when the tumors reached ~50 mm^3^. Tumors were measured using external calipers and volume calculated using the modified ellipsoidal formula: ½ (a/b^2^), where a = longest diameter and b = shortest diameter. Docetaxel was administered intraperitoneally at 4 mg/kg weekly for 3 weeks. PKC-θ peptide inhibitor was administered at 40 mg/kg daily for the duration of the experiment. All experimental procedures were performed in accordance with the guidelines and regulations accessed and approved by The Australian National University Animal Experimental Ethics Committee (Ethics ID A2014/30).

### 2.14. Flow Cytometry

Tumors were collected in cold DMEM supplemented with 2.5% FCS and finely chopped using a surgical scalpel before enzymatic dissociation using collagenase type 4 (Worthington Biochemical Corp, Lakewood, NJ, USA) to obtain single cell suspensions for flow cytometry. Cells were then stained with BD Horizon fixable viability stain 780, CD44 APC, and CD24 PE (BioLegend, San Diego, CA) and acquired using a BD LSR II instrument. Results were analyzed using FlowJo software (FlowJo, Ashland, OR, USA). 

### 2.15. RNA Sequencing

Tumors were collected in Allprotect Tissue Reagent (Qiagen, Hilden, Germany) for storage. Tumors were dissociated using the Tissue Lyser II in QIAzol lysis reagent (Qiagen) and processed using the RNeasy Microarray Tissue Mini Kit according to the manufacturer’s protocol (Qiagen). RNA was sequenced on a NextSeq500 instrument using paired-end 75 bp reads at the Ramaciotti Centre for Gene Function Analysis, University of New South Wales, Sydney.

### 2.16. RNA-Seq Bioinformatics

Reads were cleaned with *trimmomatic* (v0.36) [38] and *tagdust* (v2.33) [39] before separately mapping to the human (Hg38) and mouse (mm10) genomes (HISAT2 v2.04) [40]. Only concordantly mapped reads were kept; reads that mapped to both genomes were discarded (Picard). Gene counts were obtained using Stringtie and Ballgown [41] and TMM and library size normalized in EdgeR [42]. Principal component analysis (PCA) was performed on genes significantly different (EdgeR FDR 0.25 and >0.5 log_2_-fold change) in at least one contrast (VEH:DOC, VEH:PKC-θi, VEH:COM, DOC:COM) in R. Gene ontology enrichment (Homer) was heatmapped in R using *ggplot2*, and *bedgraph* files were prepared in Homer [43]. 

### 2.17. Synthesis of Inhibitor Peptides

PKC-θ peptide inhibitors were synthesized using automated modern solid-phase peptide synthesis and purification technology using the mild Fmoc chemistry method, for example as described in [44] and patent WO 2002/010193. Peptides were purified using automated preparative reversed-phase high-performance liquid chromatography (RP-HPLC). Fractions were analyzed using analytical RP-HPLC and mass spectrometry. Fractions of 98% purity or higher were combined to give the final product.

### 2.18. PBMC RNA Extraction and Quantitative Reverse Transcription PCR (qRT-PCR)

Isolated PBMCs were untreated or treated with 500 µM phenelzine in vitro for 10 h followed by stimulation with PMA/CaI for 4 h. Total RNA was extracted using the RNeasy Micro kit (Qiagen) according to the manufacturer’s protocols. First-strand cDNA was synthesized from 1 μg total RNA using the SuperScriptTM III First-Strand Synthesis System (Invitrogen, Thermo Fisher Scientific). Gene expression was determined by qRT-PCR with gene-specific TaqMan probes using the Applied Biosystems^®^ ViiATM 7 Real-Time PCR System (Life Technologies) and the following assay-on-demand primer/probes: *IL2* (Hs00174114), *TNF* (Hs00174128), *IFNG* (Hs00989291), and *GAPDH* (Hs99999905). 

### 2.19. NanoString nCounter Assay

RNA was isolated from tumors as in Section 2.15. The samples were then analyzed using the nCounter Single Cell PanCancer Progression CSO (115000156, NanoString Technologies) and the nCounter*^®^* FLEX analysis system (NanoString Technologies) according to the manufacturer’s instructions. In brief, 50 ng of RNA was hybridized with the reporter and capture code sets at 65 °C for 17.5 h before loading onto the prep station and analysis on the digital analyzer using the 280 fields of view setting. Data were analyzed using nSolver Analysis software. Counts for target genes were normalized using the geometric mean of housekeeping genes selected as the most stable using the geNorm algorithm. Normalized data were log2 transformed for further analyses.

### 2.20. Peptide Microarrays

Active recombinant PKC-θ was provided to JPT Peptide Technologies (Berlin, Germany) for kinase profiling on peptide microarrays. Unmodified ZEB1 peptides were chemoselectively immobilized on glass slides and incubated with kinase solution in the presence of γ-33P-ATP prior to high-resolution phosphorimaging. The spot recognition software packages GenepixPro 7.2 and ArrayPro 4.0 were used for data analysis. Peptide constructs that displayed a normalized mean signal equal to or greater than two standard deviations (SDs) above the mean were considered likely positive for phosphorylation events. Excel, R, and Python were used to determine the statistical significance of sequences and phosphorylation events. 

### 2.21. Quantification and Statistical Analysis

All statistical comparisons between sample groups were calculated using the two-tailed non-parametric Mann–Whitney test for two groups or Kruskal–Wallis test for multiple groups (GraphPad Prism) unless otherwise indicated. Data are expressed as mean ± standard error (SE). Where applicable, statistical significance is denoted by * *p* ≤ 0.05, ** *p* ≤ 0.005, *** *p* ≤ 0.0005, and **** *p* ≤ 0.0001.

## 3. Results

### 3.1. nPKC-θ Is Enriched in Immunotherapy-Resistant CTCs and Is Associated with Poor Patient Survival in Immunotherapy-Resistant Metastatic Disease

**Figure 1:**
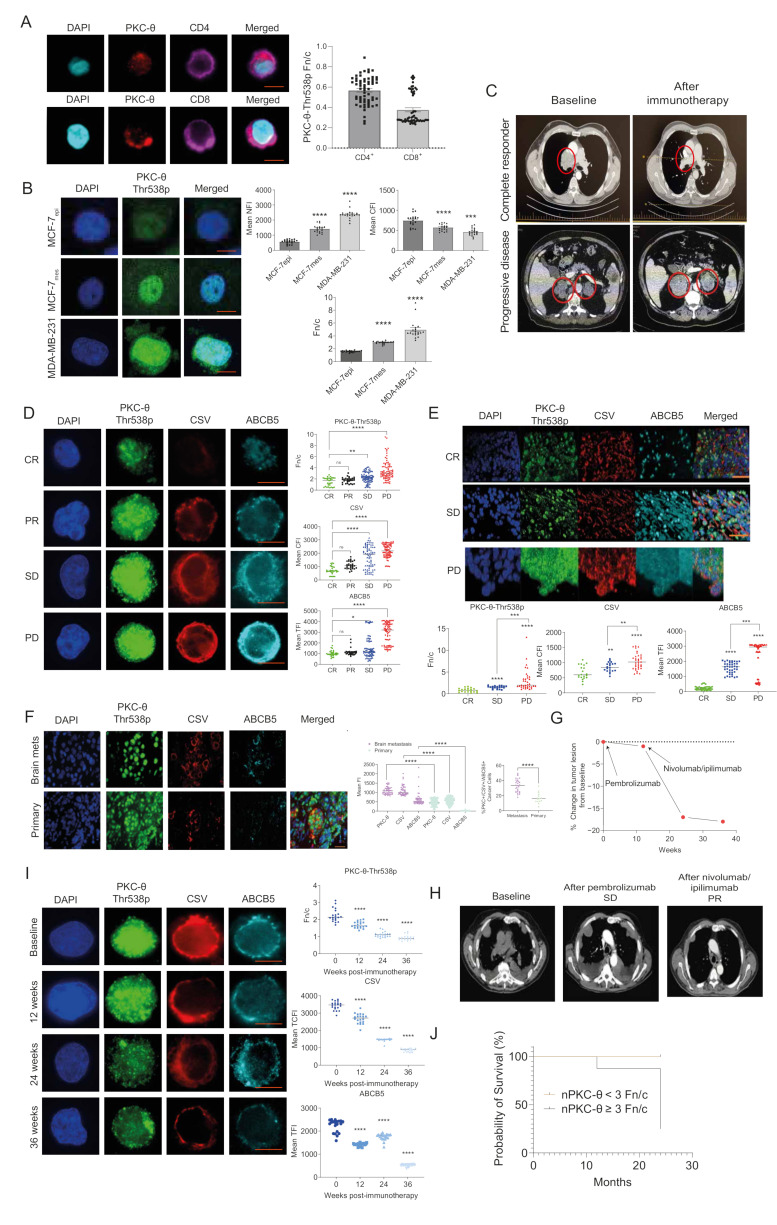
nPKC-θ signatures are enriched in CTCs and metastatic tissues and are associated with poor patient survival in immunotherapy-resistant disease. (**A**) Immunohistofluorescence analysis of PKC-θ expression in CD4^+^ and CD8^+^ T cells isolated from healthy donor liquid biopsies. Bar/dot plots show the Fn/c (nuclear to cytoplasmic ratio) of PKC-θ phosphorylated at threonine 568 (PKC-θ-Thr568p). A score below 1 indicates cytoplasmic bias. Data are from three separate patients, *n* ≥ 20 cells per patient. Representative images are shown for each dataset. PKC-θ-Thr568p (red); CD8/CD4 (purple), and DAPI (cyan) were used to visualize expression and nuclei; scale bar represents 10 µM. (**B**) Immunohistofluorescence analysis of PKC-θ expression in human MCF-7 inducible model (MCF-7-IM) and MDA-MB-231 breast cancer cells. Human MCF-7 epithelial cells (MCF-7epi) were activated with PMA to induce EMT and generate MCF-7 mesenchymal-like (MCF-7mes) breast cancer cells. Bar graphs show the mean nuclear fluorescence intensity (NFI), cytoplasmic fluorescence intensity (CFI), and Fn/c for PKC-θ-Thr568p, *n* ≥ 20 cells per group. Representative images are shown for each dataset. PKC-θ-Thr568p (green) and DAPI (blue) were used to visualize nuclei. Scale bar represents 10 µM. (**C**) Contrast-enhanced CT scans of tumors in CR and PD metastatic melanoma patients at baseline and 12 weeks after treatment with immunotherapy (nivolumab). Red circles indicate tumor lesions. Tumor lesions are reduced in CR compared with baseline, while PD shows increased tumor burden. (**D**) Dot plot quantification of PKC-θ-Thr568p, CSV, and ABCB5 fluorescence intensity in circulating tumor cells (CTCs) isolated from immunotherapy-responsive (CR, partial response (PR)) or resistant (stable disease (SD), PD) melanoma patients defined using RECIST 1.1 criteria. The Fn/c for PKC-θ-Thr568p, mean CFI for CSV, and mean TFI for ABCB5 were quantified using ASI digital pathology. Representative images are shown for each cohort (six patients were profiled per cohort, *n* ≥ 20 cells per group); scale bar represents 10 µM. (**E**) Dot plot quantification of PKC-θ-Thr568p, CSV, and ABCB5 fluorescence in FFPE sections of primary melanomas from patients (*n* = 18 patients) with CR, SD, or PD. The Fn/c for PKC-θ-Thr568p, mean CFI for CSV, and mean TFI for ABCB5 were quantified by ASI digital pathology (*n* ≥ 40 cells per patient sample, four samples per patient). Representative images for each dataset are shown, scale bar represents 30 µM. (**F**) Dot plot quantification of PKC-θ-Thr568p, CSV, and ABCB5 fluorescence intensity in FFPE sections from breast cancer brain metastases (*n* = 30 patients) and primary breast cancer biopsies (*n* = 15 patients). The Fn/c for PKC-θ-Thr568p, mean CFI for CSV, and mean TFI for ABCB5 were quantified by ASI digital pathology (*n* > 40 cells per patient sample). Representative images for each dataset are shown (top); scale bar represents 30 µM. (**G**) Percent change in tumor lesion from baseline for a single patient with metastatic melanoma (Patient D) who was resistant to first-line treatment with pembrolizumab and displayed PD (baseline, 12 weeks) as defined by RECIST 1.1 criteria but subsequently responded to second-line nivolumab and ipilimumab to show a CR (24 weeks, 36 weeks). (**H**) CT scan showing overall tumor burden in Patient D at baseline, SD, and PR (partial response). (**I**) Mesenchymal protein expression (PKC-θ-Thr568p, CSV, and ABCB5) was profiled in CTCs isolated from Patient D at 0 (baseline) and 12-, 24-, and 36-weeks post-immunotherapy. The Fn/c for PKC-θ-Thr568p, mean CFI for CSV, and mean TFI for ABCB5 were determined by ASI Digital Pathology. Representative images for each dataset are shown (*n* ≥ 20 cells per group); scale bar represents 10 µM. (**J**) Metastatic melanoma patients (*n* = 18 patients) were scored for the Fn/c of PKC-θ from four liquid biopsies over 12 months, with Fn/c categorized as <3 or ≥3 (Fn/c >1 indicates nuclear bias, whereas <1 indicates cytoplasmic bias). These patients were tracked for an additional two years (total 36 months), and their survival data are plotted as Fn/c <3 or ≥3. Statistical significance is denoted by ns (not significant), * *p* ≤ 0.05, ** *p* ≤ 0.005, *** *p* ≤ 0.0005, and **** *p* ≤ 0.0001.

We previously showed that nPKC-θ induces human breast CSCs by activating EMT transcriptional pathways [6]. Here we used a specific antibody to examine levels of PKC-θ phosphorylated at threonine 538 (Thr538p), which is associated with PKC-θ kinase activation [45] and direct tethering to chromatin of inducible CSC genes [6]. First, to assess the association between nPKC-θ and healthy or diseased states, we examined the cytoplasmic or nuclear bias of PKC-θ in CD4^+^ or CD8^+^ T cells isolated from healthy donor (HD) liquid biopsies. Analysis of the Fn/c (nuclear to cytoplasmic ratio) revealed a strong cytoplasmic bias of PKC-θ in CD4^+^ and CD8^+^ T cells (Fn/c ˂ 0.6) (Figure 1A). Consistent with our previous observations, in the EMT-inducing MCF-7 model (MCF-7-IM) [6,7], nPKC-θ expression and the nuclear to cytoplasmic PKC-θ ratio were consistently higher in mesenchymal MCF-7 cells than epithelial MCF-7 cells, while cytoplasmic PKC-θ was lower (Figure 1B). nPKC-θ was also enriched in MDA-MB-231 breast cancer cells, which express a highly mesenchymal phenotype and are enriched in CSCs [46].

To investigate the role of enriched nPKC-θ in the context of immunotherapy resistance, we next examined nPKC-θ expression in mesenchymal, stem-like CTCs isolated from immunotherapy-responsive or resistant metastatic melanoma patients using our previously described liquid biopsy protocols [7,35]. Metastatic melanoma patients were classified into complete responders (CR), partial responders (PR), stable disease (SD), or progressive disease (PD) based on their response to pembrolizumab, nivolumab, and/or ipilimumab using RECIST 1.1 criteria (Figure 1C). PKC-θ expression was significantly enriched in the nuclei of immunotherapy-resistant patients with progressive disease compared with complete or partial responders (Figure 1D and Figure S1A). CSV (cell surface vimentin), a mesenchymal marker, and ABCB5, a CSC-like marker associated with therapeutic resistance, were also more highly expressed in immunotherapy-resistant CTCs from patients with progressive disease, suggesting that this phenotype is associated with responses to immunotherapy (Figure 1D). Consistently, nuclear expression of PKC-θ, along with the expression of cytoplasmic CSV and ABCB5, were increased in primary metastatic melanomas from patients with progressive disease (Figure 1E). Furthermore, the nuclear PKC-θ^+^/CSV^+^/ABCB5^+^ phenotype represented a higher proportion of the total tumor cell population in brain metastases from breast cancer patients (median 32.4%) compared with primary breast tumors (median 13.1%), indicating an association of this phenotype with tumor metastasis (Figure 1F).

To better understand PKC-θ dynamics during the evolution of therapeutic resistance, we examined the expression of these markers in CTCs isolated throughout the clinical course of a patient with progressive metastatic melanoma who subsequently responded to second-line immunotherapy. After initial treatment with pembrolizumab, there was minimal change in tumor burden; however, tumor burden significantly reduced after combined nivolumab and ipilimumab therapy (Figure 1G,H). CTC profiling showed that the patient had a high nuclear to cytoplasmic PKC-θ ratio and mesenchymal protein expression in primary pembrolizumab-resistant CTCs at baseline, which was reversed after the patient responded to second-line treatment with nivolumab and ipilimumab (12-, 24-, and 36-weeks post immunotherapy) (Figure 1I).

We next examined the nuclear bias of PKC-θ in the context of overall patient survival in immunotherapy responsive and resistant metastatic melanoma patients over 24 months. Analysis as above of the PKC-θ Fn/c revealed that immunotherapy-responsive patients had an Fn/c value ˂3, whereas resistant patients (i.e., those with progressive disease) had an Fn/c of ≥3. While all immunotherapy-responsive patients were alive at 36 months, all but two patients with progressive disease died within 24 months (Figure 1J). This suggests that the nuclear bias of PKC-θ (Fn/c ≥ 3) is associated with poor patient survival. Super-resolution imaging (Andor spinning disc confocal microscopy) allows high-resolution visualization of proteins in different cellular compartments at the single-cell level to confirm protein localization. Using this super-resolution imaging, we interrogated the localization of PKC-θ in T cells and CTCs from responder or resistant melanoma patients. This analysis clearly demonstrated that PKC-θ was nuclear in both CTCs and CD8^+^ T-cells resistant patient samples, with antibodies targeting CSV or CD8/PD-1 clearly highlighting the cytoplasm (Figure S1B,C).

Overall, while PKC-θ expression is biased to the cytoplasm of healthy T cells, nPKC-θ is enriched in mesenchymal CSCs and CTCs, metastatic tissues, and immunotherapy-resistant disease, suggesting that its chromatin-associated functions are associated with metastatic disease.

### 3.2. ATP Competitive Catalytic PKC-θ Inhibitors Do Not Target Its Nuclear Import

**Figure 2:**
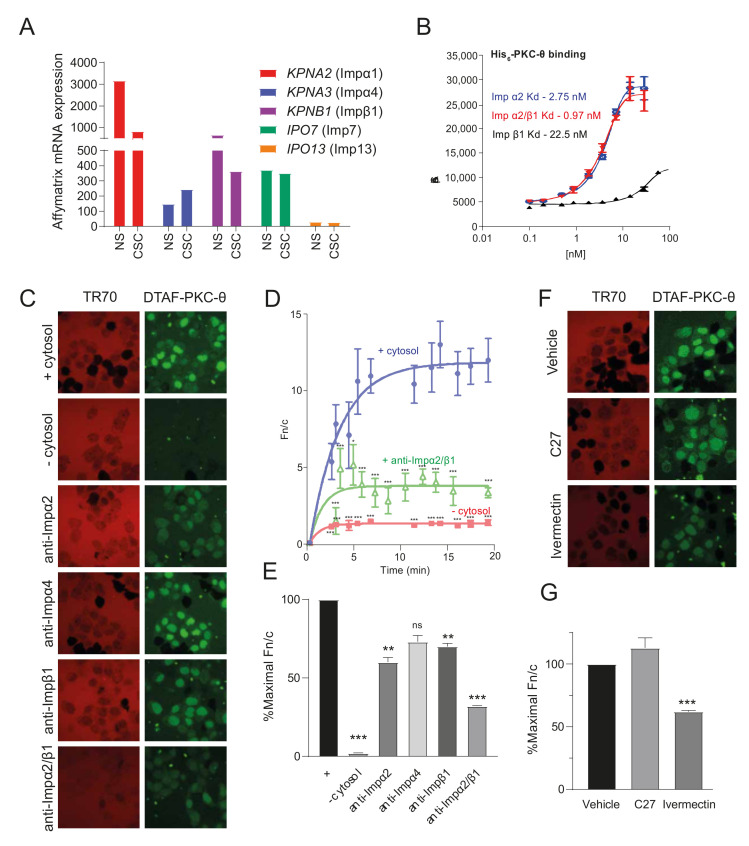
PKC-θ shows high affinity binding to the Impα/β1 heterodimer and dependence on Impα/β for nuclear accumulation in vitro. (**A**) Affymetrix microarrays previously in MCF-7 cells treated with PMA and FACS sorted into CD44^high^ and CD24^low^ cancer stem cells (CSC) and non-stem cancer cells (NS), respectively were used to profile the mRNA expression of importins [6]. Graphs plot the mRNA expression of expressed importins in NS and CSC. (**B**) The strength of binding of recombinant purified His6-PKC-θ to increasing concentrations of the indicated Imps was determined using an AlphaScreen binding assay. (**C**) Nuclear import of fluorescently labelled PKC-θ (DTAF-PKC-θ) was reconstituted in vitro in mechanically perforated HTC cells in the presence (+) or absence (−) of exogenous cytosol and an ATP regeneration system. CLSM images were acquired periodically for measurement of accumulation of DTAF-PKC-θ (green panels) into intact nuclei. Nuclear integrity was confirmed by the exclusion of Texas red-labelled 70 kDa dextran (TR70; red panels). Antibodies targeting Imps (anti-Impα2, anti-Impβ1, and anti-Impα4 or a combination of anti-Impα2 and anti-Impβ1) were also included, as indicated. Images are shown at 20 min time points. (**D**) Image analysis was performed on the photomicrographs, such as those shown in C, using ImageJ. The nuclear to cytoplasmic fluorescence ratio (Fn/c) was calculated for the indicated samples at each time point. Curve fits were determined in GraphPad Prism using an exponential one-phase association equation. Results are for the mean Fn/c ± SEM (*n* > 10). *p*-values were determined for each time point compared to the + cytosol sample using the *t*-test with Welch’s correction. *** *p* < 0.0001. (**E**) The % maximal Fn/c was determined from graphs such as those shown in D for each sample. Results represent the mean ± SEM (*n* = 3). ** *p* < 0.01, *** *p* < 0.001 compared with the no addition sample (−). (F) Nuclear import of PKC-θ was reconstituted as per (**C**) in the presence of vehicle or either the PKC-θ inhibitor C27 or the Imp α/β1-dependent nuclear transport inhibitor ivermectin. CLSM images of perforated nuclei at a 20 min time point to examine the nuclear accumulation of DTAF- PKC-θ (green panels) using TR70 (red channel) to monitor nuclear integrity. (**G**) Maximal % Fn/c for each sample was determined as per (**E**). *** *p* < 0.001 compared to no addition (−).

In transcript analyses, mRNAs for the members of the importin (Imp) superfamily of nuclear transport proteins can be detected in both non-stem cancer cells (NS) and CSCs (Figure 2A). Using a high-throughput screening approach, we determined the binding specificity of PKC-θ to candidate Imps using recombinant purified His_6_-PKC-θ and increasing concentrations of Imps α2, β1, and the α2/β1 heterodimer (Figure 2B). PKC-θ showed high affinity binding to Impα2 (kd = 2.75 nM), as well as the Impα2/β1 heterodimer (kd = 1 nM); Impβ1 bound to only a low extent. Next, the nuclear import of fluorescently labelled PKC-θ (DTAF-PKC-θ) was reconstituted in permeabilized HTC cells in the presence (+) or absence (−) of exogenous cytosol and an ATP regeneration system, with and without specific antibodies targeting Impα2, Impα4, and Impβ1 or combinations thereof. Antibodies targeting Impα2, Impα4, and Impβ1 and their combinations all reduced nuclear accumulation (lower Fn/c) of DTAF-PKC-θ (Figure 2C–E).

Given that nuclear translocation of PKC-θ is important for its role in disease states and resistance, we wanted to investigate if the highly specific ATP competitive PKC-θ inhibitor, C27, restricts its nuclear translocation. We assessed the effects of C27 inhibition on the Fn/c of DTAF-PKC-θ alongside the broad-spectrum nuclear import inhibitor ivermectin, which specifically targets Impα/β-dependent nuclear import [30,47,48]. C27 did not affect nuclear accumulation of PKC-θ in stark contrast to ivermectin (Figure 2F,G) confirming the dependence of PKC-θ nuclear import on Impα/β.

Overall, this shows that a novel class of PKC-θ inhibitor that specifically targets its nuclear import is required to target nPKC-θ activity and mesenchymal CTC and CSC phenotypes in aggressive, resistant metastatic diseases.

### 3.3. A Novel PKC-θ Peptide Inhibitor Specifically Inhibits Nuclear Translocation of PKC-θ while Preserving PKC-θ Catalytic Activity

**Figure 3:**
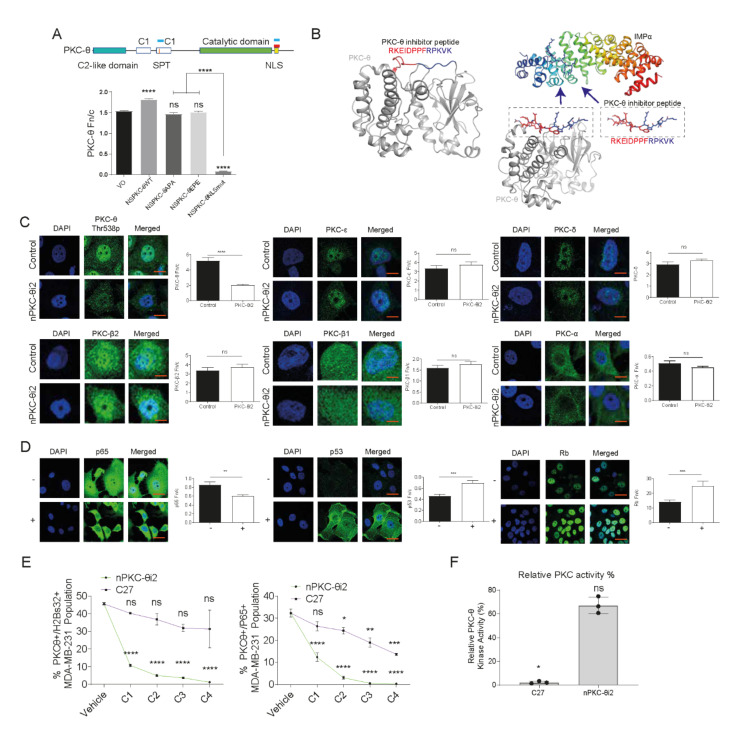
A novel PKC-θ peptide inhibitor specifically inhibits nuclear translocation of PKC-θ without affecting PKC-θ catalytic activity. (**A**) Schematic of PKC-θ depicting its domains including the canonical nuclear localization sequence (NLS) and SPT motifs. Red bars indicate location of peptide inhibitor sequences and blue bars indicate the sequence used to create PKC-θ plasmids. Graphical and schematic representation showing that PKC-θ can be targeted therapeutically. The full-length PKC-θ WT gene sequence and its mutants were used to transfect MCF-7 cells, and the localization of expressed PKC-θ was studied by confocal laser scanning microscopy. Fn/c values for each construct are shown, with significant differences between datasets indicated (*n* > 15 for each dataset). (**B**) Structure-guided design of the peptide inhibitor targeting the NLS region of PKC-θ. Left, PyMOL-generated PKC-θ based on the previously determined crystal structure (PDB 2ED) showing the region responsible for nuclear localization in red and blue. Right, structure of the nuclear import adapter IMPα (in ribbon format) bound to a cargo (in stick format) at the major binding site [50], highlighting the strategy for inhibiting nuclear localization of PKC-θ. (**C**) The nuclear localization of PKC-θ and other PKCs (PKC-β2, PKC-β1, PKC-δ, and PKC-α) were examined in the MCF-7 inducible model (IM). Representative images are displayed and the Fn/c shown. Scale bar represents 5 µm. (**D**) p65, p53, and Rb protein expression was examined in mesenchymal MCF-7 cells stimulated with PKC-θ activators PMA and TGF-β. MCF-7 cells were pretreated for 24 h with vehicle or 25 µM nPKC-θi2. Representative immunofluorescence images and Fn/c plots for MCF-7 cells treated with nPKC-θi2 are shown: − represents stimulated control; + represents stimulated samples pretreated with nPKC-θi2. Fn/c was assessed for each target protein (*n* ≥ 20 cells per group). The Mann–Whitney test was used to determine statistical significance. ns (not significant), *p* > 0.05; * *p* ≤ 0.05; ** *p* ≤ 0.01; *** *p* ≤0.001; **** *p* ≤ 0.0001. (**E**) Various concentrations of nPKC-θi2 and C27 inhibit the co-expression of PKC-θ with p65 or H2Bs32 in MDA-MB-231 cells. nPKC-θi2 concentrations: C1 = 12.5 µM, C2 = 25 µM, C3 = 50 µM, C4 = 100 µM. C27 concentrations: C1 = 1.875 µM, C2 = 3.75 µM, C3 = 7.5 µM, C4 = 15 µM. ASI digital pathology system microscopy was performed on MDA-MB-231 metastatic cancer cells probed with antibodies targeting PKC-θ and H2Bs32p with DAPI. >500 cells per group were scanned to profile the % positive population of MDA-MB-231 cells. Graphs show the % PKC-θ^+^H2Bs32^+^ population change with increasing concentrations of C27 (purple line) or nPKC-θi2 (green line). (**F**) Inhibition of recombinant PKC-θ activity (%) by C27 or nPKC-θi2 relative to untreated control using a PKC activity kit (Enzo Life Sciences).

We previously showed using a series of mutant constructs that PKC-θ localizes to the nucleus via (i) a canonical nuclear localization signal (NLS) or (ii) an SPT motif in immune cells (Figure 3A) [2,4]. In cancer cells, only the canonical NLS pathway but not the SPT pathway translocates PKC-θ to the nucleus [6]. Therefore, specific NLS motifs are active in different cell types. We therefore decided to target the nPKC-θ axis active in pathological states and not immune cells.

The human PKC-θ non-canonical bipartite NLS [49] corresponds to residues 644 to 656 (UniProt Q04759; sequence RKEID PPFRP KVK), which is 100% conserved in mice (644 to 656), rats (644 to 656), and other mammals. To further examine these NLS motifs, MCF-7 breast cancer cells were transfected with PKC-θ SPT mutants: SPT to EPE (which represents a constitutively phosphorylated serine/threonine) that induces nuclear translocation, and SPT to APA (which represents a constitutively non-phosphorylated serine/threonine) that blocks nuclear translocation [2,4]. A canonical NLS-mutated non-functional construct was also tested. We also previously demonstrated that both the SPT motif and canonical NLS function in Jurkat T cells [2], whereas only the NLS mutant prevented nuclear translocation in MCF-7 cells (Figure 3A). 

We hypothesized that molecules mimicking the canonical PKC-θ NLS would, in cancer cells, act as specific competitive inhibitors of PKC-θ translocation to the nucleus by preventing access to the importin pathway (Figure 3B). We first examined a myristoylated peptide embodying the native sequence (644–656) of PKC-θ (nPKC-θi1) and designed an optimized inhibitor based on Kosugi et al. [51] through peptide length optimization and bioinformatics analysis to identify critical residues.

We next developed an optimized myristoylated inhibitor (nPKC-θi2), which was predicted to be a more potent inhibitor of nuclear transport than the native protein sequence and have inherent cell-penetrating properties. We examined inhibition of PKC-θ nuclear translocation by nPKC-θi1 and nPKC-θi2 in MCF7-IM cells by quantitative immunofluorescence. nPKC-θi2 reduced nPKC-θ more than nPKC-θi1 (Figure S2A) and inhibited the CD44^hi^/CD24^lo^ CSC signature more than nPKC-θi1 as assessed by FACS analysis (Figure S2B). Therefore, subsequent studies were carried out with nPKC-θi2.

Previous studies have shown that all PKC isoforms show some nuclear expression [52]. Consistently, in breast cancer cells, we observed that all PKC isoforms show nuclear expression while PKC-α was largely cytoplasmic, PKC-δ, -β1, and -β2, were mostly nuclear, and PKC-θ showed the highest nuclear accumulation of all PKC isoforms (Figure S2C). To confirm the PKC-θ specificity of nPKC-θi2, MCF-7-IM cells were treated with nPKC-θi2 before being probed with antibodies specific to PKC-θ, PKC-β2, PKC-ξ, PKC-β1, PKC-δ, and PKC-α and immunofluorescence analysis. nPKC-θi2 only significantly reduced the nuclear localization of PKC-θ and had no effect on the nuclear distribution of other PKC isoforms, confirming specificity of nPKC-θi2 for PKC-θ (Figure 3C). To further probe nPKC-θi2 specificity, we developed peptide inhibitors specific to the PKC-β1, -ξ, and -δ isoforms, none of which affected the nuclear localization of PKC-θ, only the respective specific NLS as demonstrated through Fn/c analysis of immunofluorescence staining with antibodies targeting PKC-δ and -β1 isoforms (Figure S2D,E).

Next, we investigated the impact of nPKC-θi2 on downstream targets of PKC-θ. nPKC-θi2 reduced nuclear localization of p65 and increased nuclear expression of p53 and the retinoblastoma (Rb) tumor suppressor protein (Figure 3D). Chromatin-associated PKC-θ has previously been shown to directly phosphorylate the transcription factor p65 and globally phosphorylate chromatinized H2Bser32 in mesenchymal breast CSCs [5]. Using varying concentrations of catalytic inhibitor C27 and nPKC-θi2 in MDA-MB-231 cells, nPKC-θi2 reduced the proportion of PKC-θ^+^/H2Bser32^+^ and PKC-θ^+^/p65^+^ cells to a much greater extent than C27-treated MDA-MB-231 cells relative to untreated MDA-MB-231 cells (Figure 3E).

To determine if nPKC-θi2 maintains cytoplasmic PKC-θ catalytic activity, which is important in normal T-cell function, catalytic activity was assessed using recombinant PKC-θ in the presence of nPKC-θi2 or catalytic inhibitor C27. nPKC-θi2 did not affect the catalytic activity of PKC-θ compared with the C27 catalytic inhibitor control (Figure 3F). 

Our novel inhibitor, nPKC-θi2, is distinct from traditional PKC-θ inhibitors that directly target the catalytic domain and exclusively targets the nPKC-θ axis while preserving the PKC-θ catalytic activity required for normal cytoplasmic signaling.

### 3.4. nPKC-θ Induces CSC Signatures and Mesenchymal Pathways in Metastatic and Resistant Cancer Cell Lines

**Figure 4:**
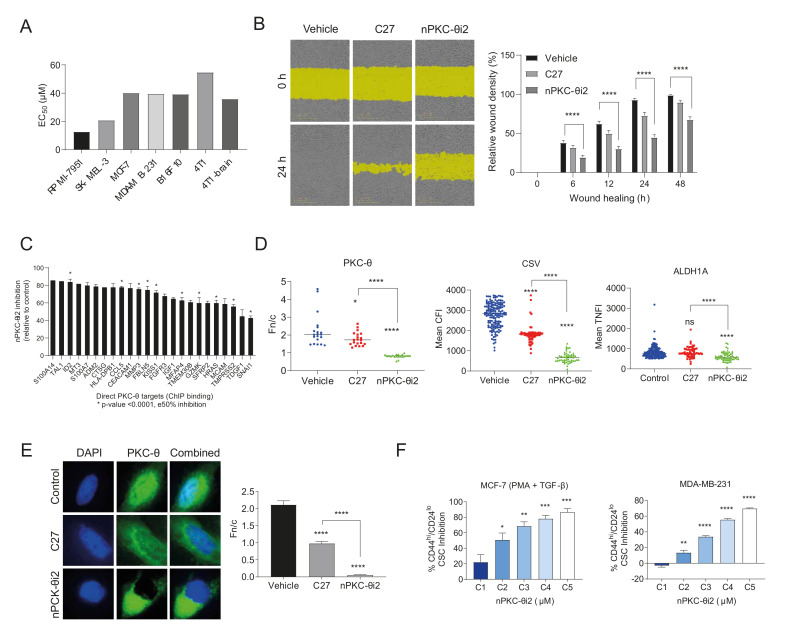
nPKC-θi2 targets CSC signatures and mesenchymal pathways in metastatic and resistant cancer cell lines. (**A**) WST proliferation assay in melanoma (RPMI-7951, SK-MEL-3), breast cancer (MDA-MB-231), and immunotherapy-resistant (4T1, 4T1 brain clone, and B16F10) cancer cell lines. Cells were treated with nPKC-θi2 for 72 h before addition of WST reagent. Absorbance was measured at 450 nm. (**B**) Scratch wound assay in MDA-MB-231 breast cancer cells treated with vehicle, C27 (7.5 µM), or nPKC-θi2 (25 µM). Wound healing images were acquired by real-time imaging using the IncuCyte Zoom live cell analysis system every 6 h for 24 h. Relative wound density (%) was analyzed using IncuCyte Zoom software. One-way ANOVA was used to determine statistical significance. ns (not significant) *p* > 0.05; * *p* ≤ 0.05; ** *p* ≤ 0.01; *** *p* ≤0.001; **** *p* ≤ 0.0001. (**C**) RNA isolated from MDA-MB-231 murine tumors treated with nPKC-θi2 was analyzed using the NanoString pan-cancer panel. * indicates direct PKC-θ binding targets determined by overlaying NanoString data with ChIP sequencing data from PKC-θ enriched MDA-MB-231 samples. (**D**) Dot plot quantification of PKC-θ-Thr568p, CSV, and ALDH1A fluorescence intensity in the 4T1 TNBC brain cancer clone treated with vehicle, C27 (5 µM), or nPKC-θi2 (25 µM). The Fn/c for PKC-θ-Thr568p, mean CFI for CSV, and mean NFI for ALDH1A were determined by immunohistofluorescence analysis. *n* ≥ 20 cells per group. (**E**) Dot plot quantification of PKC-θ-Thr568p in MDA-MB-231 brain cancer clone cells treated with vehicle, C27 (5 µM) or nPKC-θi2 (25 µM). The Fn/c for PKC-θ-Thr568p was determined by immunohistofluorescence analysis. The Mann–Whitney test was used to determine statistical significance. ns (not significant) *p* > 0.05; * *p* ≤ 0.05; ** *p* ≤ 0.01; *** *p* ≤ 0.001; **** *p* ≤ 0.0001. (**F**) FACS plot of % CD44^hi^/CD24^lo^ CSC inhibition in mesenchymal-like MCF-7 cells activated with PMA and TGF-β and MDA-MB-231 breast cancer cells treated with nPKC-θi2 1 µM (C1), 5 µM (C2), 25 µM (C3), 50 µM (C4), and 100 (C5) µM relative to their respective untreated control cells.

Given that high PKC-θ expression has been shown to enhance the proliferation and migration of cancer cells [53,54], we next investigated the effect of nPKC-θi2 inhibition on breast cancer, melanoma, and immunotherapy-resistant cancer cell lines. We determined the half maximal effective concentration (EC_50_) of nPKC-θi2 in several cell lines. nPKC-θi2 inhibited melanoma (RPMI-7951, SK-MEL-3) and breast cancer (MCF-7, MDA-MB-231) cell lines, as well as cell lines highly resistant to immunotherapy (B16F10, 4T1, 4T1 brain metastasis) (Figure 4A and Figure S3A–C). Consistent with previous reports, inhibition of PKC-θ suppressed MDA-MB-231 cell migration, but nPKC-θi2 inhibited migration to a greater extent than C27 relative to vehicle (Figure 4B).

Next, we identified direct gene targets of nPKC-θ from cancer-associated pathways using RNA samples isolated from MDA-MB-231 murine tumors treated with nPKC-θi2 using the NanoString pan-cancer panel and overlaying the results with chromatin immunoprecipitation (ChIP) sequencing data [6] from MDA-MB-231 enriched for PKC-θ (Figure 4C). Bioinformatics analysis revealed that nPKC-θ directly regulates genes involved with mesenchymal, metastatic CSC-like signatures and tumor markers such as S100A14 (a marker associated with metastasis). Next, to determine if both cytoplasmic and nPKC-θ induce mesenchymal signatures in immunotherapy-resistant cancers, we examined the expression of metastasis markers in the 4T1 brain metastasis cell line. Inhibition with nPKC-θi2 further reduced the expression of nPKC-θ, CSV, and ALDH1 compared to treatment with C27 (Figure 4D). Consistent with our previous results, catalytic inhibition with C27 reduced nPKC-θ expression; however, nPKC-θ was further reduced by nPKC-θi2 in MDA-MB-231 triple-negative breast cancer cells (TNBC, brain cancer clone) relative to vehicle control (Figure 4E). Given that nPKC-θi2 targets mesenchymal pathways, we also analyzed the CD44^hi^/CD24^lo^ CSC population in activated MCF-7 and MDA-MB-231 cells to determine if nPKC-θ inhibited CSCs. nPKC-θi2 reduced the CD44^hi^/CD24^−^ CSC population in both cell lines (Figure 4F).

Together, these data suggest that nPKC-θ induces mesenchymal signatures in cancer cells and increases proliferation, migration, and CSC formation and that its inhibition reverses these phenotypes.

### 3.5. Inhibition of nPKC-θ Reduces Mesenchymal Signatures in Primary Tumors and CTCs In Vivo

**Figure 5:**
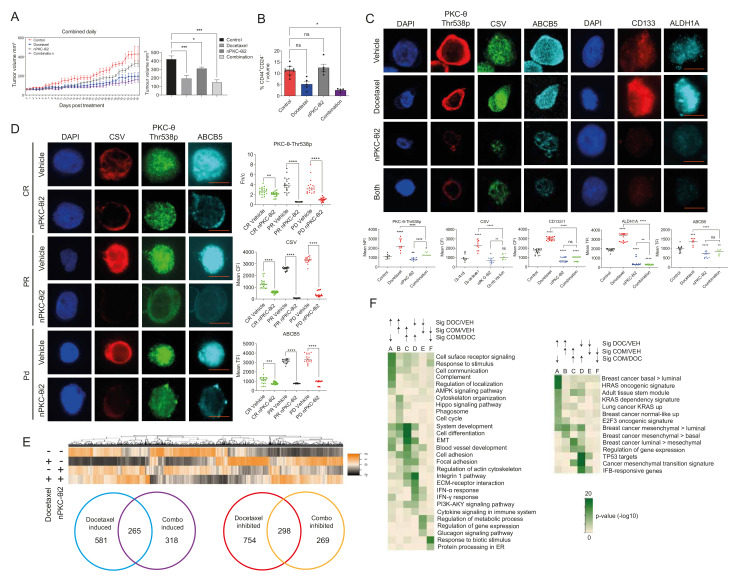
Impact of the novel PKC-θ inhibitor on tumors in a TNBC xenograft model and CTCs from melanoma patients. (**A**) Tumor volume in MDA-MB-231 mouse-bearing tumors treated with vehicle control, docetaxel (4 mg/kg), nPKC-θi2 (40 mg/kg), or both (docetaxel given 3 times, 1 week apart and nPKC-θi2 given daily for 5 weeks). Tumor volumes were measured daily for each mouse. Each data point represents a single mouse (*n* = 4 mice per group). (**B**) Percent CD44^hi^/CD24^lo^ CSC cells in total tumor in the MDA-MB-231 mouse model. (**C**) Immunofluorescence microscopy of tumor cells from MDA-MB-231 TNBC mice treated with nPKC-θi2 in combination with docetaxel showing that nPKC-θi2 inhibits the fluorescence intensity of PKC-θ and key stem cell niche markers CD133, ALDH1A, and ABCB5 and mesenchymal marker CSV. (**D**) CTCs were isolated from melanoma patient liquid biopsies (CR = complete response, PR = partial response, PD = progressive disease) and were pre-clinically treated with either vehicle control or nPKC-θi2. Samples were fixed and immunofluorescence microscopy performed on these cells with primary antibodies targeting CSV, PKC-θ, and ABCB5. Representative images for each dataset are shown. Graph represents the TCFI values for CSV, NFI for PKC-θ, and TFI for ABCB5 measured using ImageJ to select the nucleus minus background (*n* ≥ 20 cells/sample). (**E**) Heatmap of tumor transcriptomes using all significant genes together with a Venn diagram comparison of genes induced by docetaxel/combination therapy or inhibited by docetaxel/combination therapy relative to vehicle control and the overlap between these groups. (**F**) Heat map of enriched pathways in gene sets induced relative to vehicle control with comparison of geneset pathways induced by docetaxel (DOC) or docetaxel and nPKC-θi2 (COM). Statistical significance is denoted by ns (not significant), * *p* ≤ 0.05, ** *p* ≤ 0.005, *** *p* ≤ 0.0005, and **** *p* ≤ 0.0001.

To investigate the effect of nPKC-θi2 in vivo, MDA-MB-231 tumor-bearing mice were treated with vehicle, docetaxel (4 mg/kg), nPKC-θi2 (40 mg/kg), or both (docetaxel and nPKC-θi2). In the MDA-MB-231 xenograft model, primary tumor burden was controlled by single treatment with docetaxel or in combination with nPKC-θi2 compared with vehicle (Figure 5A). Combination treatment with both agents together further suppressed tumor volume and resulted in greater inhibition of CD44^+^/CD24^−^ CSCs per tumor volume compared to single treatment with docetaxel or nPKC-θi2 (Figure 5B).

We have shown that targeting nPKC-θ inhibits the mesenchymal, stem-like signatures in breast cancer cell lines. In the MDA-MB-231 xenograft model, treatment with nPKC-θi2 alone or in combination with docetaxel reduced the expression of nPKC-θ, CSV, ABCB5, CD133/1, and ALDH1 in nude mouse tumors compared with vehicle (Figure 5C). As in our previous studies, treatment with docetaxel alone significantly increased the expression of these metastasis-associated proteins in surviving tumor cells [35]. To further investigate the role of nPKC-θ in regulating metastatic phenotypes in a clinical setting, CTCs from melanoma patients were treated ex vivo with nPKC-θi2. Consistently, nPKC-θi2 significantly reduced the expression of nPKC-θ and the metastatic markers CSV and ABCB5 in all CTC cohorts (CR, PR, PD) compared with vehicle controls (Figure 5D).

Overall, the data demonstrate that nPKC-θ is overexpressed in mesenchymal, stem-like cancer cells and induced in chemotherapy-resistant cancer cells to orchestrate a CSC-like mesenchymal signature. Specifically targeting the nPKC-θ axis is critical for inhibiting the mesenchymal, CSC-like, resistance signature in metastatic and immunotherapy-resistant cancers.

### 3.6. Targeting nPKC-θ Reduces the Chemotherapy-Induced Mesenchymal Signature on Tumor Cell Transcriptome

Given that chemotherapy is part of the standard of care for TNBC, we next investigated the impact of nPKC-θ inhibition in combination with chemotherapy at the transcriptional level by transcriptomic profiling of MDA-MB-231 xenografts treated with vehicle, docetaxel (4 mg/kg), nPKC-θi2 (40 mg/kg), or their combination. Mapping the paired-end RNA-seq reads to both the human and mouse genomes allowed us to separate the tumor (human) transcriptome from the tumor microenvironment (mouse) transcriptome. As expected, most reads mapped to the human genome, with end library sizes for the tumor environment around 5–10.5 M (Figure S4A). Consistent with the tumor sizes, the docetaxel, nPKC-θi2, and combination-treated mice had a greater (15–23%) proportion of reads from the tumor microenvironment than vehicle controls (12–13%) (Figure S4B). Both tumor and tumor microenvironment expression values were normalized to their respective library size. The PCA plot demonstrates clusters of samples based on their similarity and that the treatment groups cluster together and are distinct from the vehicle control (Figure S4C).

Docetaxel induced more changes in the tumor transcriptome (FDR 0.25, > 0.5 log_2_-fold) than either nPKC-θi2 or combination therapy (Figure S4D). In total, 848 genes were transcriptionally induced in docetaxel-treated tumors compared with vehicle (Figure S4D,E). Total of 581 genes were induced by docetaxel treatment alone (Figure 5E), and this treatment group was significantly enriched for genes in the HRAS oncogenic signature [55] and in basal or mesenchymal type than luminal signatures [56] and for biological processes such as response to stimulus, cell communication, signaling, and regulation of localization (Figure 5E,F). Of the 580 genes significantly induced by combination therapy (Figure S4D,E), 318 were not significantly induced by docetaxel alone and only in the combination treatment group (Figure 5E). 

In contrast, a total of 1052 genes were transcriptionally downregulated in docetaxel-treated tumors (Figure 5E and Figure S4D,E). Of these downregulated genes, 754 genes were only inhibited by docetaxel treatment alone and were not decreased in tumors that received both docetaxel and nPKC-θi2 combination treatment (Figure 5E). The docetaxel-inhibited gene group was enriched for IFNA response [57], cancer EMT signature [58], integrin 1 pathway, and collagen metabolic processes and included genes such as *FN1, TNFSF10, IFIT3, IFI44*, *ARHGEF4/9*, several collagen and serpin genes, and the transcription factors *FOXS1, ZEB2, BHLHE41, RUNX2*, and *RORB* (Figure 5E,F). Interestingly, the combination induced group had genes in common with the docetaxel-inhibited group and was enriched for genes involved in differentiation, cell adhesion, locomotion, and EMT (Figure 5E,F). However, despite enrichment for some EMT genes such as collagens, the combination-induced group was enriched for luminal more than mesenchymal genes and enriched for genes decreased in basal tumors (SMID) such as *CST3, PRLR* (prolactin receptor), *MUC1, CELSR1, ABCC3, TC9, EC14L2, FLRT3, SNED1, FBXL7, NO1, ST6GALNAC2*, and *PCDH1* (Figure 5E,F). Other genes in this set included *TNFSF9*, *ID1/3*, and transcription factors such as *FOSL1*, *HES1*, and *BHLHE40* (Figure 5E,F).

Total of 298 genes were significantly downregulated independently of nPKC-θi2 in combination-treated tumors (Figure 5E). This set of genes also included those involved in regulation of metabolic processes and transcription factors such as *NR3C1* (GR), *NR2C2*, and *RORA* (Figure 5E,F). Furthermore, of the total 567 genes (Figure 5E) inhibited by combination treatment, a total of 269 were decreased only by combination treatments and were unaffected by docetaxel monotherapy (Figure 5E). These genes were enriched for the response to biotic stimulus and included several heat shock genes, three histone H2B variants, and *CXCR4* (Figure 5E,F). Together, these data show that chemotherapy induces a mesenchymal tumor signature that was reduced by simultaneous treatment with nPKC-θi2.

### 3.7. PKC-θ Is Enriched in the Nuclei of CD8^+^ T Cells Isolated from Stage IV Metastatic Cancers

**Figure 6:**
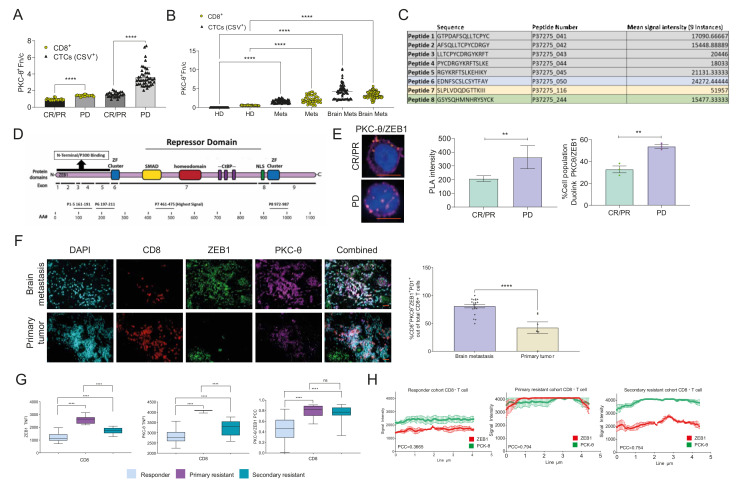
PKC-θ is enriched in the nuclei of dysfunctional CD8+ T cells isolated from stage IV metastatic cancers. (**A**) Quantification of PKC-θ-Thr568p Fn/c in CD8+ T cells or CSV+ CTCs isolated from immunotherapy-responsive (complete response, CR; partial response PR) or resistant (PD, progressive disease) melanoma patients defined using RECIST 1.1 criteria. The Fn/c for PKC-θ-Thr568p was quantified by ASI digital pathology and ImageJ-Fiji (*n* ≥ 20 cells per group). (**B**) Quantification of PKC-θ-Thr568p Fn/c in CD8+ T cells or CSV+ CTCs isolated from healthy donors (HD), stage IV metastatic breast cancer patients (Mets), or stage IV breast cancer patients with brain metastases (Brain Mets). The Fn/c for PKC-θ-Thr568p was quantified by ASI digital pathology and ImageJ-Fiji (*n* ≥ 40 cells per group). (**C**) The amino acid sequence of ZEB1 indicating the top eight peptides for peptide phosphorylation by PKC-θ. The top peptide sequences are displayed in a table with the mean signal intensity (2 SD above the mean was considered a positive phosphorylation event). (**D**) Top peptides positive for phosphorylation and their overlap with the ZEB1 amino acid sequence as well as the structure of ZEB1, adapted from [3]. (**E**) Duolink^®^ proximity ligation assay (PLA) for PKC-θ and ZEB1 in CD8+PD1+ T cells isolated from immunotherapy-resistant or responder melanoma patients. Representative images are shown for PKC-θ/ZEB1, scale bar represents 10 µM. Graphs represent the PLA signal intensity of the Duolink^®^ assay; data represent *n* ≥ 100 cells/sample. Graphs plot the percentage of PLA signal positive cells out of total cells for (A). Data represent *n* ≥ 100 cells/sample. (**F**) FFPE sections from primary breast cancers (*n* = 6 patients, >500 cells counted per patient) or breast cancer brain metastases (*n* = 20 patients, >500 cells counted per patient) were processed for high-resolution microscopy using the BondRX platform. FFPE sections were fixed and immunofluorescence microscopy performed probing with primary antibodies targeting CD8, PKC-θ (T53p), and ZEB1 with DAPI. Plots represent the % population of CD8+ T cells positive for PKC-θ and ZEB1 out of total CD8+ T cells. Example images are shown with 20 µM scale bar. (**G**) CD8+ cells were isolated from melanoma patient liquid biopsies (responder = complete response (CR) or resistant, where primary = primary resistance, secondary = secondary resistance, PD = progression of disease) and stimulated with phorbol 12-myristate 13-acetate (PMA) and calcium ionophore (CI) and pre-clinically screened with either vehicle control or nPKC-i2. Samples were then fixed and immunofluorescence microscopy performed with primary antibodies targeting ZEB1, PKC-θ, and CD8. Representative images for each dataset are shown in Figure S5E. Graph represents the mean TNFI for PKC-θ and ZEB1 measured using ImageJ to select the nucleus minus background (*n* > 20 cells/sample). (**H**) Plot profiles for each cohort for ZEB1 and PKC-θ are also depicted (red = ZEB1, green = PKC-θ) with the Pearson correlation coefficient (PCC) used to quantify colocalization between fluorophore-tagged proteins indicated and plotted. −1 = inverse of colocalization; 0 = no colocalization; +1 = perfect colocalization. Statistical significance is denoted by ns (not significant), ** *p* ≤ 0.005 and **** *p* ≤ 0.0001.

Given that we identified a new role for nPKC-θ in immunotherapy resistance, we next sought to understand the contribution of nPKC-θ to the anti-tumor immune response in metastatic cancers. Consistent with our results in CTCs, nPKC-θ expression was higher in CD8^+^ T cells isolated from patients with immunotherapy-resistant stage IV metastatic melanoma than those with immunotherapy-responsive disease (Figure 6A and Figure S5A). Interestingly, nPKC-θ expression was also low in CD4^+^ human Jurkat T cells and CD8^+^ T cells isolated from immunotherapy responders with metastatic melanoma (Figure S5B). This suggests that nPKC-θ may, in part, contribute to immunotherapy resistance through its action in CD8^+^ T cells in addition to its role in CTCs. nPKC-θ was also enriched in the nuclei of CTCs and CD8^+^ T cells from patients with stage IV metastatic breast cancers with brain metastasis or patients with TNBCs (Figure 6B). PKC-θ was also enriched in the tumor microenvironment (TME) of brain cancer lesions in the EO771 metastatic brain cancer model and in the nuclei of CD8^+^ T cells within this TME (Figure S5C). The H&E stained sections of the EO771 brain metastasis model are depicted in Figure S5D.

### 3.8. PKC-θ Forms a Repressive Complex with ZEB1 in the Nuclei of CD8^+^ T Cells Isolated from Metastatic Cancer Patients

Given that nPKC-θ is enriched in CD8^+^ T cells from patients with immunotherapy-resistant tumors, we next wanted to investigate the role of nPKC-θ in the regulation of dysfunctional T cells. T-cell dysfunction is often a hallmark of cancer and an underlying mechanism of immunotherapy resistance [24,25,59]. ZEB1 is a key repressive transcription factor in EMT that induces PD-L1 expression in the tumor microenvironment, leading to immune suppression [60,61]. Furthermore, ZEB1 expression is also downregulated when CD8^+^ T cells are activated and regulates effector and memory CD8^+^ T cell fate [62], suggesting that ZEB1 may be linked to a dysfunctional CD8^+^ T-cell signature. 

We previously showed that nPKC-θ is also a key regulator of nuclear ZEB1 in breast cancer cells [4]. We therefore wanted to understand if PKC-θ interacts with ZEB1 in CD8^+^ T cells. To determine if PKC-θ directly phosphorylates ZEB1, we performed a peptide microarray to determine the top ZEB1 sequences for PKC-θ phosphorylation (Figure 6C). The sequence with the highest signal intensity (peptide 7) overlapped the SMAD binding domain, which is crucial for controlling ZEB1 transcriptional activity (Figure 6D) [63]. The third highest signal (peptide 8) overlapped the N-terminal zinc finger (ZF) cluster, which is critical for ZEB1 DNA-binding activity [63], and is adjacent to the NLS domain, suggesting that it may influence nuclear localization (Figure 6D). Together, this suggests that PKC-θ may directly phosphorylate ZEB1 to regulate its DNA binding activity and nuclear localization.

To further investigate the relationship between nPKC-θ and dysfunctional T-cell transcription, we assessed interactions between PKC-θ and ZEB1 in PD-1^+^/CD8^+^ T cells isolated from immunotherapy-responsive or -resistant melanoma patients. Using a proximity ligation assay, which detects close interactions between two target proteins, PKC-θ was shown to exist in proximity to ZEB1 in the nuclei of immunotherapy-resistant PD-1^+^/CD8^+^ T cells but not immunotherapy-responsive T cells derived from melanoma patients (Figure 6E). Furthermore, PD-1^+^/CD8^+^ T cells co-expressing both nPKC-θ and ZEB1 represented 80.0% of the total CD8^+^ T-cell population in breast cancer brain cancer metastases compared to 54.7% in primary breast metastases, suggesting that nPKC-θ may interact with ZEB1 as part of an exhaustion signature important for tumor progression and metastasis (Figure 6F). Matching H&E-stained sections are shown in Figure S6A.

We next examined ZEB1 and PKC-θ expression in CD8^+^ T cells isolated from liquid biopsies of metastatic stage IV melanoma patients stratified based on RECIST v1.1 into responder, primary resistance, or secondary resistance (resistant after an initial positive response to immunotherapy). PKC-θ and ZEB1 were enriched in primary and secondary resistance CD8^+^ T-cell nuclei compared with patients responding to immunotherapy (Figure 6G), with plot-profile and correlation analysis showing that PKC-θ and ZEB1 co-localized in primary and secondary resistant disease compared with responding patients (Figure 6H). Overall, our data demonstrate that PKC-θ is enriched in dysfunctional PD1^+^/CD8^+^ T cells derived from resistant melanoma patients and that, in dysfunctional PD1^+^/CD8^+^ T cells, PKC-θ forms a nuclear complex with ZEB1 that may potentially drive the CD8^+^ T-cell exhaustion phenotype.

### 3.9. Inhibition of nPKC-θ Inhibits Dysfunctional ZEB1/PKC-θ Nuclear Complex and Induces Cytokine Expression in CD8^+^ T Cells

**Figure 7:**
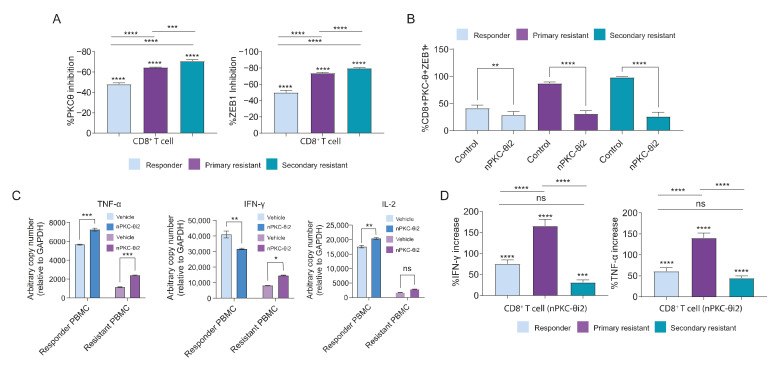
nPKC-θi2 disrupts the nuclear ZEB1/PKC-θ complex and induces cytokine production in CD8+ T cells. (**A**) Graphs depicting the % inhibition or induction based on protein expression were also plotted for each protein target relative to untreated sample. (**B**) Percent of PKC-θ+/ZEB1+/CD8+ T cells in samples isolated from melanoma patients responsive (PR/CR) or primary/secondary resistant (PD) to immunotherapy. CD8+ T cells were treated with nPKC-θi2 before activation ex vivo with PMA/ionomycin. (**C**) Gene expression of key effector cytokines IL2, IFNG, and TNFA in PBMCs isolated from resistant and responder patients either treated with vehicle control or nPKC-θi2 before activation ex vivo with PMA/ionomycin. (**D**) Protein expression of TNF-α and IFN-γ in CD8+ T cells isolated from primary or secondary resistant or responder patient liquid biopsies and treated with nPKC-θi2. Graphs show the % CD8+ increase in expression of TNF-α or IFN-γ in CD8+ T cells stimulated with PMA/ionomycin in addition to treatment with nPKC-θi2. One-way ANOVA was used to compare groups, where ns (not significant), **** *p* < 0.0001, *** *p* < 0.001, ** *p* < 0.01, and * *p* < 0.05.

To further elucidate the functional role of PKC-θ and ZEB1 in CD8^+^ T cells, we inhibited nPKC-θ with nPKC-θi2 in CD8^+^ T cells isolated from immunotherapy-responsive (CR/PR) or resistant (PD) melanoma patients. Ex vivo treatment with nPKC-θi2 significantly reduced both nuclear expression of the ZEB1/PKC-θ complex and the population of CD8^+^ cells positive for ZEB1/PKC-θ (Figure 7A,B) and significantly increased mRNA expression of *IL-2* in responder PBMCs and *TNFA* in both responder and resistant PBMCs (Figure 7C). nPKC-θi2 also significantly increased *IFNG* transcription in resistant PBMCs but decreased transcription in PBMCs isolated from responder patients (Figure 7C). To validate upregulation of these effector cytokines at the protein level, CD8^+^ T cells isolated from liquid biopsies of immunotherapy-responsive or -resistant melanoma patients were treated with nPKC-θi2 in vitro before activation with PMA and ionomycin (Figure 7D). nPKC-θi2 treatment significantly increased the population of CD8^+^ T cells expressing IFN-γ and TNF-α in both immunotherapy-responsive and -resistant CD8^+^ T cells relative to vehicle control. To determine the impact of PKC-θ inhibition on normal T cells, PBMCs isolated from HDs were treated ex vivo with nPKC-θi2 or C27 and viability determined by FACS. nPKC-θi2 and C27 did not significantly reduce viability of the total PBMC population, CD4^+^ T cells, or CD8^+^ T cells compared with untreated vehicle controls (Figure S6B).

Therefore, PKC-θ^+^/ZEB1^+^ co-localization was greater in CD8^+^ T cells from patients with resistant disease compared with responders, and ex vivo inhibition of CD8^+^ T cells with nPKC-θi2 decreased this signature and enhanced cytokine production in both responder and resistant CD8^+^ T cells. Directly targeting nPKC-θ is likely to reinvigorate T-cell immune responses and reverse the dysfunctional T-cell phenotype in metastatic, immunotherapy-resistant disease.

## 4. Discussion

PKC-θ is a serine/threonine kinase belonging to a growing class of kinases with dual roles as both cytoplasmic signaling kinases that transiently phosphorylate their substrates and chromatin-associated kinases that stably interact with the epigenome. We previously showed that chromatin-associated PKC-θ functions as a critical molecular switch of the CSC epigenome, leading to the induction of key EMT transcriptional pathways in human breast CSC lines [6]. Here we suggest that PKC-θ’s nuclear chromatin-associated role is a feature of disease states, particularly cancer metastasis and resistance, while its cytoplasmic signaling activity is conserved in healthy, functional T cells. In this study, we extend our previous work and show that nPKC-θ is enriched in mesenchymal CTCs and dysfunctional CD8^+^ T cells in aggressive metastatic cancers, namely TNBC brain metastases and immunotherapy-resistant metastatic melanoma. To target enriched nPKC-θ, we designed a novel PKC-θ-specific peptide inhibitor that disrupts nPKC-θ but maintains its catalytic activity. Targeting nPKC-θ inhibited mesenchymal signatures in CTCs and allowed re-expression of key effector cytokines in CD8^+^ T cells in mouse tumor models and patient-derived cancer and immune cells. Overall, we show for the first time that nuclear-enriched PKC-θ mediates immunotherapy resistance via its nuclear activity in mesenchymal CTCs and dysfunctional CD8^+^ T cells.

Cytoplasmic PKC-θ is a feature of normal CD4^+^ and CD8^+^ T cells in healthy individuals, while mesenchymal cancer cell lines and CTCs isolated from patients resistant to immunotherapy show high nuclear bias of PKC-θ expression. Cytoplasm-biased PKC-θ is redistributed into the nucleus of breast cancer cells on induction of the mesenchymal phenotype. Furthermore, nPKC-θ is highly expressed in CSC-enriched TNBC cells. This is consistent with previous studies showing that PKC-θ is highly expressed in estrogen receptor-negative (ER-) human breast tumors and TNBC cell lines but not ER^+^ breast tumors or breast cancer cell lines [53,64] and further supports our previous work showing that nPKC-θ regulates inducible EMT transcription programs in breast cancer cells [6]. 

We used a Canadian-developed gold standard digital pathology platform (ASI) to investigate PKC-θ in expression in cells and tissues. This digital pathology platform is particularly useful for quantifying nuclear immunofluorescence staining in patient samples derived from liquid biopsies (even with low cell numbers) or tissue biopsies. Digital pathology has major advantages over other protein assays such as immunoblotting, which does not have the sensitivity to detect protein in limited patient samples nor spatial resolution. Digital pathology analysis allows easy quantification of the subcellular localization of proteins and, importantly, this platform allows expression analysis in individual cell types to determine population dynamics [34,35,36]. Using this digital pathology platform, we show that CTCs isolated from patients with advanced melanoma resistant to immunotherapy are enriched in nuclear PKC-θ. We also show that key metastatic proteins (CSV and ABCB5) are co-expressed alongside nPKC-θ in CTCs and tumor metastases. This is consistent with our previous findings showing that CSV and ABCB5 are highly expressed in CTCs isolated from melanoma patients [35]. This profile was also highly expressed in brain metastases in breast cancer patients, further suggesting that nPKC-θ plays a critical role in metastatic progression. Furthermore, CTC profiling of a single patient with progressive melanoma demonstrated that increased nPKC-θ and mesenchymal protein expression was reversed after the patient responded to second-line immunotherapy. Importantly, high nuclear localization of PKC-θ was predictive of immunotherapy resistance and poor patient survival in metastatic melanoma. High nPKC-θ expression has also been associated with disease recurrence and poor survival in patients with oral squamous cell carcinoma [65]. Overall, the nuclear-enriched PKC-θ represents a unique and specific therapeutic target in patients with aggressive metastatic disease including advanced melanoma, TNBC, and metastatic brain cancer, that are also likely be resistant to immunotherapy.

Given the specificity of nPKC-θ for pathological states, we investigated the therapeutic efficiency of directly targeting nPKC-θ rather than its catalytic activity by designing a novel peptide inhibitor mimicking the NLS (nPKC-θi2) to target the nPKC-θ axis. nPKC-θi2 is a non-catalytic PKC-θ inhibitor that mimics the NLS of PKC-θ and directly competes with importin-α and importin-α/β subunits to prevent translocation of PKC-θ from the cytoplasm to the nucleus. Treatment with nPKC-θi2 reduced nuclear localization of PKC-θ and direct targets of PKC-θ in mesenchymal phenotype-induced breast cancer cells. In addition, restricting nPKC-θ translocation inhibited the proliferation of breast cancer, melanoma, and immunotherapy-resistant cancer cell lines and breast cancer cell migration in mesenchymal CSC-enriched breast cancer cell lines. Targeting the nPKC-θ axis reduced the CD44^hi^/CD24^lo^ CSC population in mesenchymal breast cancer cell lines, consistent with previous studies showing that high PKC-θ expression results in aberrant cell proliferation, migration, and invasion [54,64,66].

We recently showed that exclusively targeting the nuclear activity of LSD1, a critical epigenetic enzyme in breast CSC formation, more efficiently inhibits the stem-like mesenchymal signature than traditional FAD-specific LSD1 catalytic inhibitors [35]. Similarly, our nPKC-θ-specific inhibitor reduced high nuclear accumulation of PKC-θ and global phosphorylation of H2Bser32 in mesenchymal phenotype-induced breast cancer cells to greater extent than the catalytic inhibitor C27. In contrast to C27, nPKC-θi2 maintained PKC-θ’s catalytic activity, suggesting that PKC-θ can still participate in cytoplasmic signaling in normal, healthy T cells. We hypothesize that exclusively targeting nuclear localization of PKC-θ and protecting its catalytic activity would enhance the inhibition of immunotherapy-resistant cells while limiting the adverse toxic effects that may occur through global targeting of PKC-θ’s catalytic activity.

We also show for the first time that nPKC-θ induces mesenchymal signatures in immunotherapy-resistant settings in both CTCs and TNBC xenografts. In tumor cells isolated from TNBC xenografts, targeting the nPKC-θ axis significantly reduced the expression of nuclear localized PKC-θ in addition to several key CSC-like mesenchymal markers including CSV, ABCB5, ALDH1, and CD133/1. In MDA-MB-231 TNBC xenografts, mesenchymal marker expression was further reduced in combination with chemotherapy, consistent with previous studies showing that inhibiting PKC-θ enhances the chemosensitivity of epithelial breast cancer and TNBC cell lines [67]. We observed similar responses at the transcript level in MDA-MB-231 tumors treated with combined nPKC-θi2 and chemotherapy. The expression of nPKC-θ and key resistance markers were also significantly reduced in CTCs isolated from responder or resistant melanoma patients and treated ex vivo with nPKC-θi2. Overall, these data show that nPKC-θ in combination with immunotherapy may improve responses in immunotherapy-resistant metastatic cancer patients. Therefore, when targeting these pathways in cancer patients, a triple and sequential combination of nPKC-θ and chemotherapy or immunotherapy is likely to be required to completely overcome resistance and recurrence.

In addition to its role in CTCs, we show that nPKC-θ contributes to immunotherapy resistance by inducing T-cell dysfunction. Dysfunctional CD8^+^ T cells are a hallmark of aggressive cancers with immunotherapy resistance. Consistent with our CTC data, PKC-θ was also enriched in the nuclei of dysfunctional CD8^+^ T cells isolated from immunotherapy-resistant melanoma patients. Furthermore, PKC-θ was expressed at low levels in the nuclei of CD4^+^ human Jurkat T cells and CD8^+^ T cells isolated from immunotherapy-responsive metastatic melanoma patients. We previously showed that nPKC-θ is also a key regulator of nuclear ZEB1 in breast cancer cells [4]. Here we extend these findings and show that nPKC-θ is an upstream regulator of the ZEB1 exhaustion signaling network in immunotherapy-resistant dysfunctional CD8^+^ T cells. nPKC-θ seems to form a repressive complex with ZEB1, a key repressive transcription factor in EMT activation, that is disrupted by inhibition of nPKC-θ. Furthermore, nPKC-θi2 also induced the expression of effector cytokines in PBMCs isolated from immunotherapy-resistant melanoma patients, suggesting that PKC-θ induces the dysfunctional CD8^+^ T-cell phenotype in immunotherapy-resistant tumors. Finally, ex vivo treatment with nPKC-θi2 increased the expression of TNF-α and IFN-γ in CD8^+^ T cells from responder and resistant patients with advanced melanoma, consistent with our previous work showing that PKC-θ inhibition regulates the expression of these cytokines in human memory CD4^+^ T cells [5]. Interestingly, while TNF-α and IL-2 transcription increased, IFN-γ transcription decreased in immunotherapy-responsive PMBCs after in vitro treatment with nPKC-θi2. We have previously shown that nuclear PKC-θ modulates gene expression post-transcriptionally by binding to the promoters of micro(mi)RNAs that regulate T-cell repressor proteins such as ZEB1 crucial for cytokine regulation [4]. Therefore, nPKC-θ may be regulating IFN-γ expression at the post-transcriptional level via miRNAs rather than at the chromatin level.

Many studies have now shown that different classes of epigenetic inhibitors can reverse or overcome immunotherapy resistance in tumors through upregulation of chemokine expression, the antigen processing and presentation machinery, and immune checkpoint molecules, ultimately enhancing immune responses in patients receiving immunotherapy [68]. Therefore, it will be important to rationally combine PKC-θ inhibitors with chemotherapy and immunotherapy in vitro and in vivo to further investigate the role of PKC-θ in overcoming immunotherapy resistance. In this study, we validated our nPKC-θ inhibitor in the context of the TME using human immunotherapy-responsive and -resistant patient samples with intact immune systems. We also showed that PKC-θ is enriched in the TME of brain cancer lesions in the EO771 metastatic brain cancer model. Future studies should also aim to investigate the biological significance of nPKC-θi2 in immune-competent mouse models such as 4T1 murine breast cancer. Genome-wide ChIP sequencing will also be important to identify other downstream targets of nPKC-θ regulating the mesenchymal and exhaustion signaling networks in cancer cells and CD8^+^ T cells. Furthermore, it will also be important to investigate the expression of nPKC-θ in other immune cells such as NK cells, Tregs, and macrophages in addition to CD8^+^ T cells in patients with immunotherapy-resistant disease.

## 5. Conclusions

Overall, we have identified for the first time that nPKC-θ is enriched in CTCs and CD8^+^ T cells in metastatic cancer patients, especially in those with immunotherapy-resistant cancers. nPKC-θ induces mesenchymal signatures and inhibits effector CD8^+^ T-cell responses. We propose that enrichment of nPKC-θ in dysfunctional CD8^+^ T cells decorates the nucleus with regulatory proteins that induce this dysfunctional program, including ZEB1. To target this complex and overcome T-cell exhaustion and CSC burden, PKC-θ inhibitors that directly target chromatin-associated PKC-θ are required. Inhibition of nPKC-θ in combination with standard therapies such as chemotherapy or immunotherapy may improve therapeutic responses in difficult-to-treat cancers such as metastatic melanoma or metastatic TNBC while preserving the normal, homeostatic functions of cytoplasmic PKC-θ.

## Data Availability

Data are contained within the article or supplementary material and from the corresponding author on reasonable request.

## Supplementary Materials

The following supporting information can be downloaded at: https://www.mdpi.com/article/10.3390/cancers14061596/s1, Figure S1. (A) Depicts bar graph quantification of PKC-θ-Thr568p fluorescence intensity in circulating tumor cells (CTCs) isolated from immunotherapy responsive (CR, partial response (PR)) or resistant (stable disease (SD), PD) melanoma patients defined using RECIST 1.1 criteria. The mean NFI for PKC-θ-Thr568p was quantified by ASI digital pathology. (B) Representative image of melanoma responder or resistant CTCs imaged using the Andor WD Revolution Inverted Spinning Disk microscopy system. Cells were permeabilized and immunostained with antibodies targeting CSV and PKC-θ; DAPI (blue) was used to visualize nuclei. Scale bar is 10 µm. (C) Representative image of melanoma responder or resistant CD8^+^ T cells imaged using the Andor WD Revolution Inverted Spinning Disk microscopy system. Cells were permeabilized and immunostained with antibodies targeting CD8, PD-1, and PKC-θ; DAPI (blue) was used to visualize nuclei. Scale bar is 10 µm. Figure S2. (A) Immunohistofluorescence analysis of PKC-θ expression in mesenchymal MCF-7 inducible model (MCF7-IM) cells inhibited with nPKC-θi1 or nPKC-θi2. (B) The CD44^hi^/CD24^lo^ signature assessed by FACS in mesenchymal MCF-IM cells inhibited with nPKC-θi1 or nPKC-θi2. (C) Representative immunofluorescence microscopy pictures and Fn/c plots for MCF-7-IM cells labelled with antibodies targeting PKC-δ, -β2, -α, -β1, and -θ. Fn/c was used to determine the nuclear localization for *n* ≥ 20 cells. (D) The impact of the nPKC-θi2 on the nuclear localization of PKC-θ and other PKCs (PKC-β2, PKC-ξ, PKC-β1, PKC-δ, and PKC-α). Representative immunofluorescence microscopy photomicrographs and the plot of Fn/c for MCF-7 cells treated with peptide inhibitors targeting PKC-β1, PKC-δ, and PKC-ε, where ST represents mesenchymal MCF-7-IM cells. Fn/c was used to determine the impact on nuclear localization for *n* ≥ 20 cells. The Mann–Whitney non-parametric *t*-test was used to compare groups where **** *p* < 0.0001, *** *p* < 0.001, ** *p* < 0.01, and * *p* < 0.05. Scale bar represents 5 µm. (E) The impact of nPKC-β1 or nPKC-δ1 on the nuclear localization of PKC-β1 or PKC-δ. Representative immunofluorescence microscopy photomicrographs and plot of Fn/c for MCF-7 cells treated with peptide inhibitors targeting PKC-β1 and PKC-δ in mesenchymal MCF-7-IM cells. Fn/c was used to determine the impact on nuclear localization for *n* ≥ 20 cells. The Mann–Whitney non-parametric *t*-test was used to compare groups where **** *p* < 0.0001, *** *p* < 0.001, ** *p* < 0.01, and * *p* < 0.05. Scale bar represents 5 µm. Figure S3. WST proliferation assay on (A) melanoma, (B) breast cancer, and (C) immunotherapy-resistant cell lines showing the EC_50_ of nPKC-θi2 (PKC pep2) on the proliferation of these cell lines at 72 h post-inhibition. Figure S4. (A,B) Library sizes of RNA-seq reads uniquely mapping to the human (tumor) or mouse (tumor environment) genome. Library sizes from EdgeR. Graph shows averages from 3–4 mice +/− SEM. (C) PCA cluster plot of genes induced by different treatment groups docetaxel, nPKC-θi2 and combination treatment. (D) Table of significantly (FDR 0.25, > log_2_ 0.5-fold change) differentially expressed genes in the various contrasts of vehicle (Veh), docetaxel (DOC), nPKC-θi2 (PKCi), or docetaxel and PKC-θi2 combination (COM) treated mice. (E) Summary of the upregulation/downregulation of genesets with different treatments. Figure S5. (A) CTCs from liquid biopsies from healthy donors (HD) and breast cancer patients: ER/PR+/-/HER2+ or TNBC were labelled with antibodies targeting PKC-θ T538p, and the Fn/c for each cohort determined by immunofluorescence analysis. (B) Immunohistofluorescence analysis of PKC-θ expression in human MCF-7 (NS/ST), MDA-MB-231 breast cancer cells, Jurkat T cells, and CD8+ T cells from an immunotherapy responsive patient. Human MCF-7 epithelial cells (MCF-7epi) were activated with PMA to induce EMT and generate MCF-7 mesenchymal-like (MCF-7mes) breast cancer cells. Bar graphs show the nuclear to cytoplasmic ratio (Fn/c) for PKC-θ phosphorylated at threonine 568 (PKC-θ-Thr568p) (*n* ≥ 20 cells per group). Representative images are shown for each dataset (left). PKC-θ-Thr568p (green); DAPI (blue) was used to visualize nuclei, scale bar represents 10 µM. (C) Representative FFPE tumor sections from the E0771 brain metastasis mouse model treated with a cancer vaccine or vehicle control. Samples were stained using tyramide staining for PKC-θ and CD8 and imaged using the ASI Digital pathology platform to automatically count population dynamics with automatic thresholding. Graph plots of the % infiltration of total CD8^+^ T cells into scanned tumor sections relative to total tumor cells and the graph plot of the proportion of CD8^+^ T cells positive for nuclear PKC-θ (nPKCθ^+^) and PD-1. One-ANOVA was used to calculate significant differences, ns = no significance, * *p* < 0.05, ** *p* < 0.005, *** *p* < 0.0005, **** *p* < 0.0001. (D) Representative H&E-stained sections from mice treated with either control or a cancer vaccine matching the immunofluorescence staining in (C). (E) Depicts example images of CD8 cells isolated from melanoma patient liquid biopsies [Responder = complete response (CR) or resistant (primary = primary resistance, secondary = secondary resistance, PD = progression of disease)] stimulated with PMA/CI and pre-clinically screened with either vehicle control or nPKC-θi2. Samples were then fixed and immunofluorescence microscopy was performed on these cells with primary antibodies targeting ZEB1, PKC-θ, and CD8. Figure S6. (A) Representative H&E-stained sections of a brain metastasis from a stage IV breast cancer patient matching the immunofluorescence staining in Figure 6F. (B) FACS plot showing % cell viability of total PBMCs, CD4^+^ T cells, and CD8^+^ T cells isolated from healthy donors and treated ex vivo with C27 or nPKC-θi2.
