# Supplementary material for: Selective Targeting of Protein Kinase C (PKC)-θ Nuclear Translocation Reduces Mesenchymal Gene Signatures and Reinvigorates Dysfunctional CD8+ T Cells in Immunotherapy-Resistant and Metastatic Cancers
Source: Cancers (Basel). 2022 Mar 21;14(6):1596. doi: 10.3390/cancers14061596 (PMC8946217; doi:10.3390/cancers14061596)
Supplement: Supplementary file 1 [file cancers-14-01596-s001.zip › cancers-1610536.pdf]

Supplementary Materials

Selective Targeting of Protein Kinase C (PKC)-θ Nuclear Translocation Reduces Mesenchymal Gene Signatures and Reinvigorates Dysfunctional CD8⁺ T Cells in Immunotherapy-Resistant and Metastatic Cancers

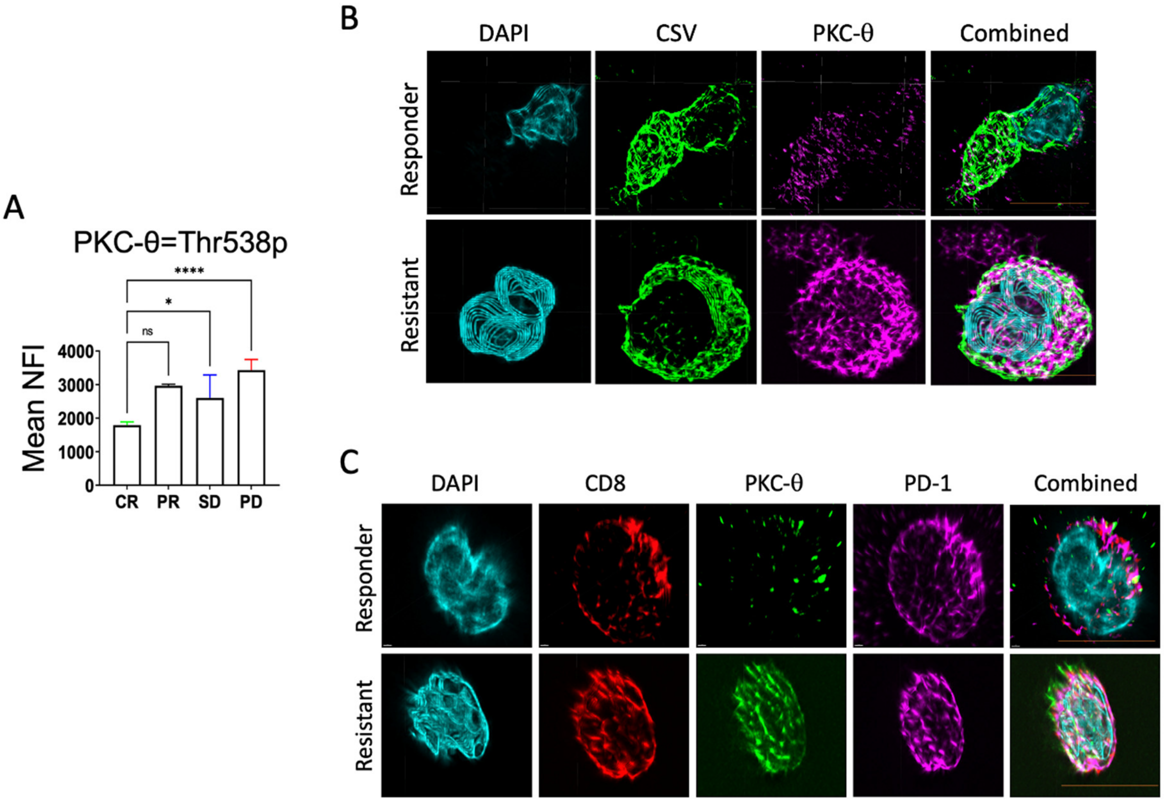

Figure S1. (**A**) Depicts bar graph quantification of PKC-θ-Thr568p fluorescence intensity in circulating tumor cells (CTCs) isolated from immunotherapy responsive (CR, partial response (PR)) or resistant (stable disease (SD), PD) melanoma patients defined using RECIST 1.1 criteria. The mean NFI for PKC-θ-Thr568p was quantified by ASI digital pathology. (**B**) Representative image of melanoma responder or resistant CTCs imaged using the Andor WD Revolution Inverted Spinning Disk microscopy system. Cells were permeabilized and immunostained with antibodies targeting CSV and PKC-θ; DAPI (blue) was used to visualize nuclei. Scale bar is 10 µm. (**C**) Representative image of melanoma responder or resistant CD8⁺ T cells imaged using the Andor WD Revolution Inverted Spinning Disk microscopy system. Cells were permeabilized and immunostained with antibodies targeting CD8, PD-1, and PKC-θ; DAPI (blue) was used to visualize nuclei. Scale bar is 10 µm. **** $p < 0.0001$, *** $p < 0.00$ and * $p < 0.05$.

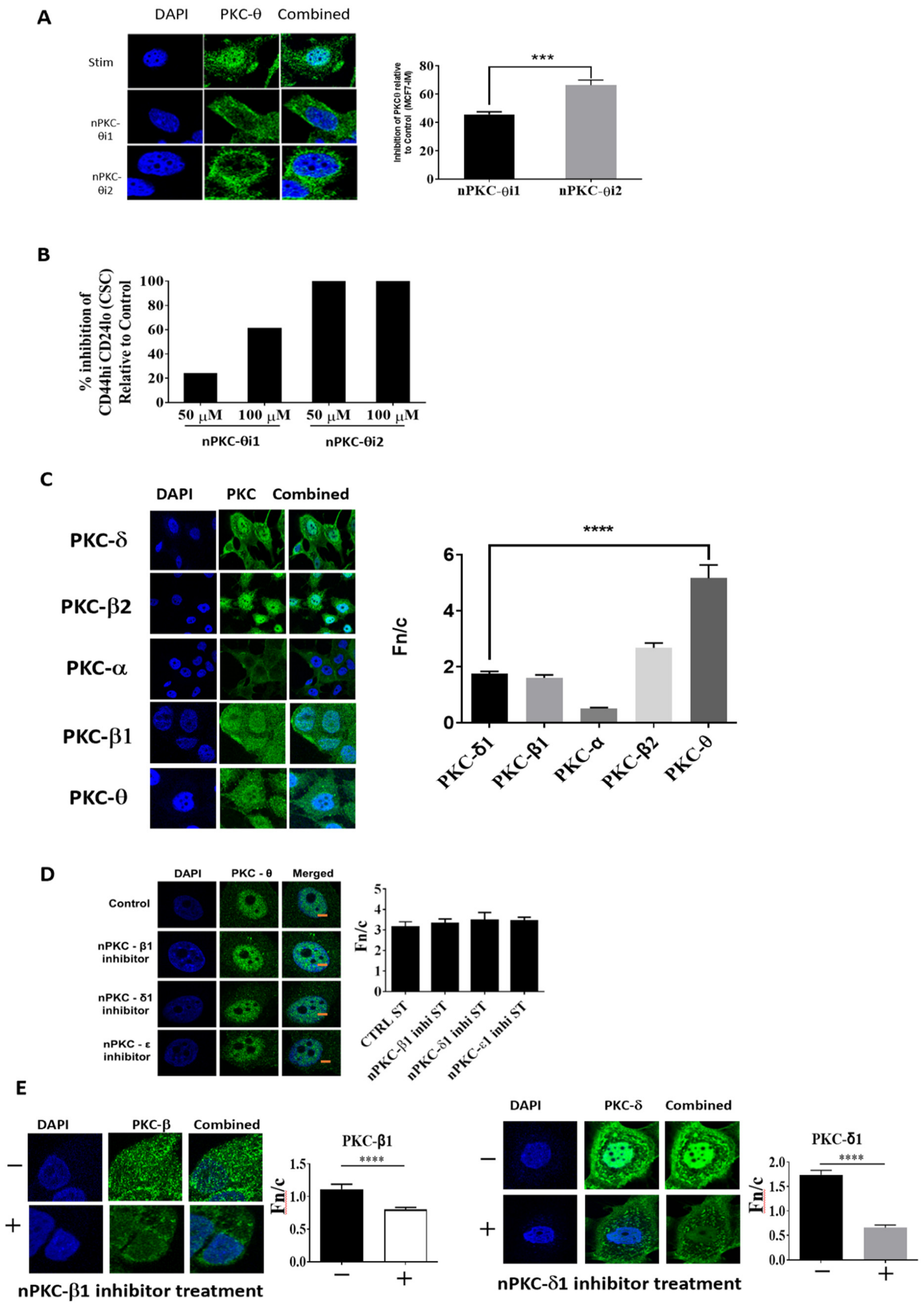

Figure S2. (**A**) Immunohistofluorescence analysis of PKC-θ expression in mesenchymal MCF-7 inducible model (MCF7-IM) cells inhibited with nPKC-θi1 or nPKC-θi2. (**B**) The CD44hi/CD24lo signature assessed by FACS in mesenchymal MCF-IM cells inhibited with nPKC-θi1 or nPKC-θi2. (**C**) Representative immunofluorescence microscopy pictures and Fn/c plots for MCF-7-IM cells labelled

with antibodies targeting PKC-δ, -β2, -α, -β1 and -θ. Fn/c was used to determine the nuclear localization for n ≥ 20 cells. (**D**) The impact of the nPKC-θi2 on the nuclear localization of PKC-θ and other PKCs (PKC-β2, PKC-ξ, PKC-β1, PKC-δ, and PKC-α). Representative immunofluorescence microscopy photomicrographs and the plot of Fn/c for MCF-7 cells treated with peptide inhibitors targeting PKC-β1, PKC-δ, and PKC-ε, where ST represents mesenchymal MCF-7-IM cells. Fn/c was used to determine the impact on nuclear localization for n ≥ 20 cells. The Mann-Whitney non-parametric t-test was used to compare groups where **** $p < 0.0001$, *** $p < 0.001$, ** $p < 0.01$, and * $p < 0.05$. Scale bar represents 5 µm. (**E**) The impact of nPKC-β1 or nPKC-δ1 on the nuclear localization of PKC-β1 or PKC-δ. Representative immunofluorescence microscopy photomicrographs and plot of Fn/c for MCF-7 cells treated with peptide inhibitors targeting PKC-β1 and PKC-δ in mesenchymal MCF-7-IM cells. Fn/c was used to determine the impact on nuclear localization for n ≥ 20 cells. The Mann-Whitney non-parametric *t*-test was used to compare groups where **** $p < 0.0001$, *** $p < 0.001$, ** $p < 0.01$, and * $p < 0.05$. Scale bar represents 5 µm.

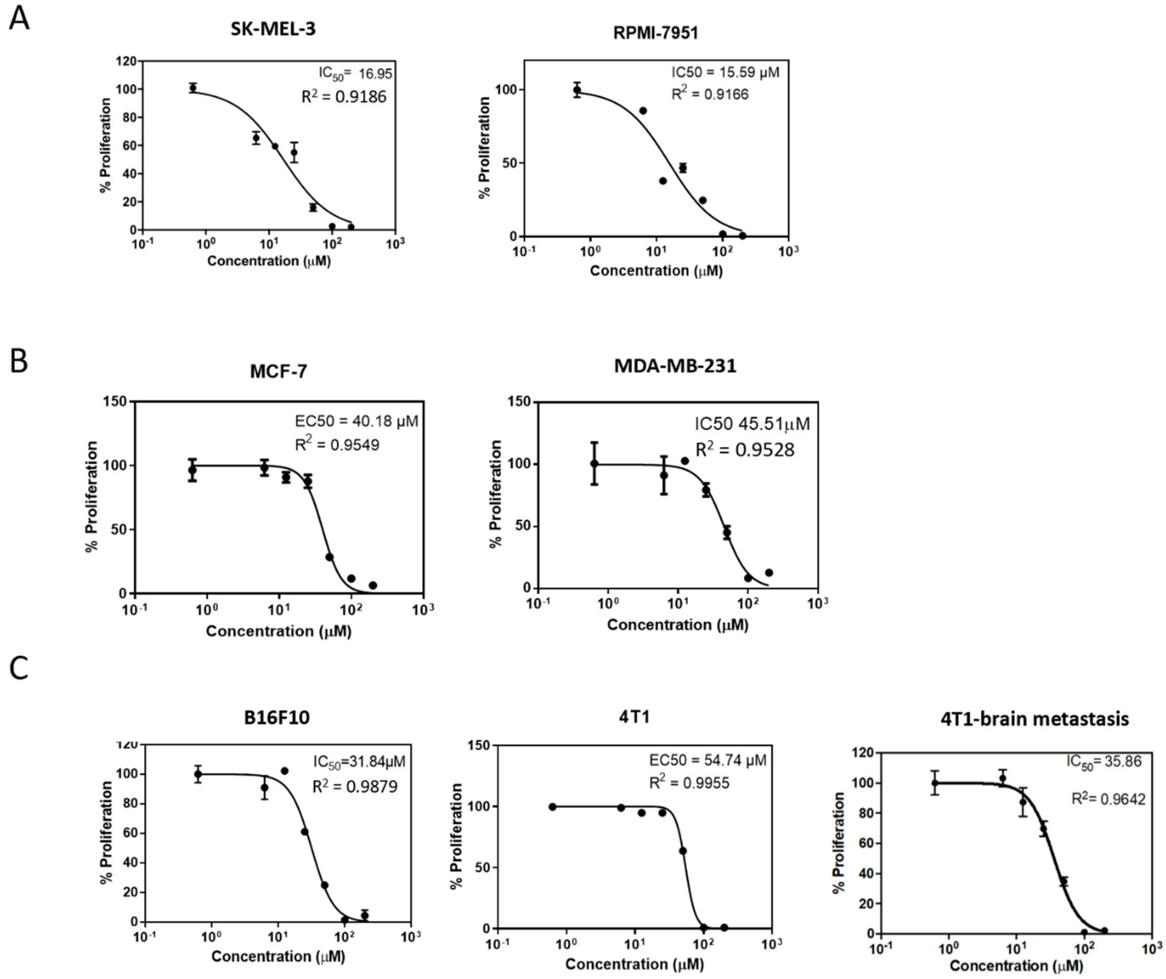

Figure S3. WST proliferation assay on (**A**) melanoma, (**B**) breast cancer, and (**C**) immunotherapy resistant cell lines showing the EC_{50} of nPKC-θi2 (PKC pep2) on the proliferation of these cell lines at 72 h post-inhibition.

A

		Reads.		Percent	
id	group	Human	Mouse	Human	Mouse
A327/VEH1	VEH	36,040,184	4,953,782	88	12
A328/VEH2	VEH	42,989,344	5,824,060	88	12
A330/VEH3	VEH	36,887,021	5,544,209	87	13
B331/DOC1	DOC	36,795,433	8,933,673	80	20
B333/DOC2	DOC	36,502,762	8,056,290	82	18
B335/DOC3	DOC	35,812,658	9,729,614	79	21
E347/PKC1	PKC	38,753,766	6,867,726	85	15
E348/PKC2	PKC	31,174,750	7,383,206	81	19
E349/PKC3	PKC	40,992,807	9,103,805	82	18
H361/COM1	COM	36,951,079	8,711,370	81	19
H362/COM2	COM	35,902,041	10,492,268	77	23
H363/COM3	COM	30,061,554	8,539,897	78	22
H364/COM4	COM	43,142,059	8,716,646	83	17

B

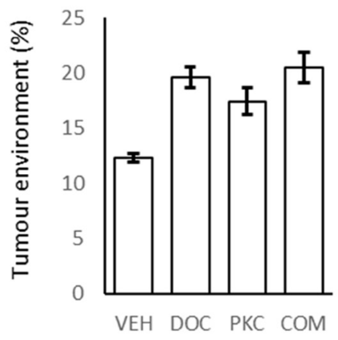

C

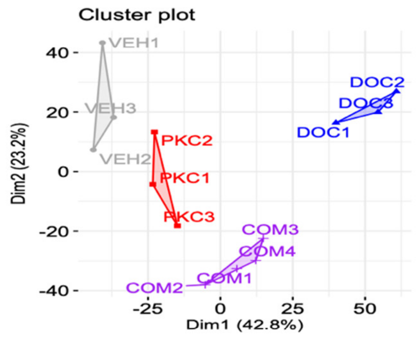

D

A	B	UP (B>A)	DOWN (A>B)
Veh	DOC	848	1052
Veh	PKCi	54	39
Veh	COM	580	567
DOC	COM	654	453

E

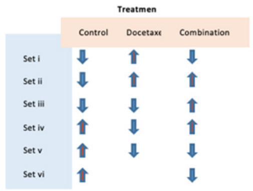

Figure S4. (**A–B**) Library sizes of RNA-seq reads uniquely mapping to the human (tumor) or mouse (tumor environment) genome. Library sizes from EdgeR. Graph shows averages from 3-4 mice +/- SEM. (**C**) PCA cluster plot of genes induced by different treatment groups docetaxel, nPKC-θi2 and combination treatment. (**D**) Table of significantly (FDR 0.25, > $\log_2$ 0.5-fold change) differentially expressed genes in the various contrasts of vehicle (Veh), docetaxel (DOC), nPKC-θi2 (PKCi), or docetaxel and PKC-θi2 combination (COM) treated mice. (**E**) Summary of the upregulation/downregulation of genesets with different treatments.

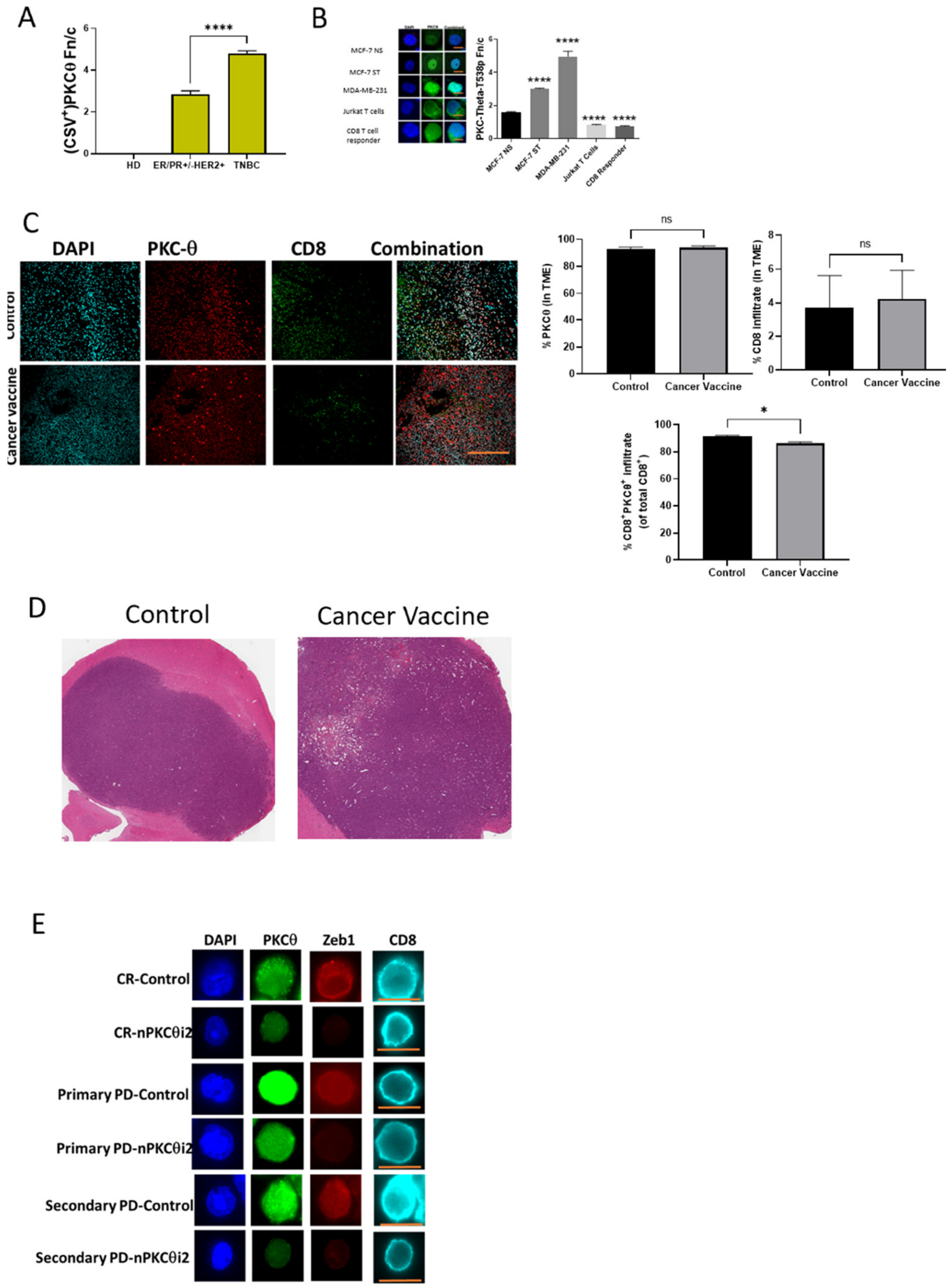

Figure S5. (**A**) CTCs from liquid biopsies from healthy donors (HD) and breast cancer patients: ER/PR+/-/HER2+ or TNBC were labelled with antibodies targeting PKC-θ T538p, and the Fn/c for each cohort determined by immunofluorescence analysis. (**B**) Immunohistofluorescence analysis of PKC-θ expression in human MCF-7 (NS/ST), MDA-MB-231 breast cancer cells, Jurkat T cells, and CD8+ T cells from an immunotherapy responsive patient. Human MCF-7 epithelial cells (MCF-7epi) were activated with PMA to induce EMT and generate MCF-7 mesenchymal-like (MCF-7mes) breast cancer cells. Bar graphs show the nuclear to cytoplasmic ratio (Fn/c) for PKC-θ phosphorylated at threonine 568 (PKC-θ-Thr568p) ($n \geq 20$ cells per group). Representative images are shown for each dataset (left). PKC-θ-Thr568p (green); DAPI (blue) was used to visualize nuclei, scale bar represents 10 μM. (**C**) Representative FFPE tumor sections from the E0771 brain metastasis mouse model treated with a cancer vaccine or vehicle control. Samples were stained using tyramide staining for PKC-θ and CD8 and imaged using the ASI Digital pathology platform to automatically count population dynamics with automatic thresholding. Graph plots of the % infiltration of total $CD8^+$ T cells into scanned tumor sections relative to total tumor cells and the graph plot of the proportion of $CD8^+$ T

cells positive for nuclear PKC-θ (nPKCθ+) and PD-1. One-ANOVA was used to calculate significant differences, ns = no significance, * < 0.05, ** < 0.005, *** < 0.0005, **** < 0.0001. (**D**) Representative H&E-stained sections from mice treated with either control or a cancer vaccine matching the immunofluorescence staining in (**C**). (**E**) Depicts example images of CD8 cells isolated from melanoma patient liquid biopsies [Responder = complete response (CR) or Resistant (primary = primary resistance, secondary = secondary resistance, PD = progression of disease)] stimulated with PMA/CI and pre-clinically screened with either vehicle control or nPKC-θi2. Samples were then fixed and immunofluorescence microscopy was performed on these cells with primary antibodies targeting ZEB1, PKC-θ, and CD8.

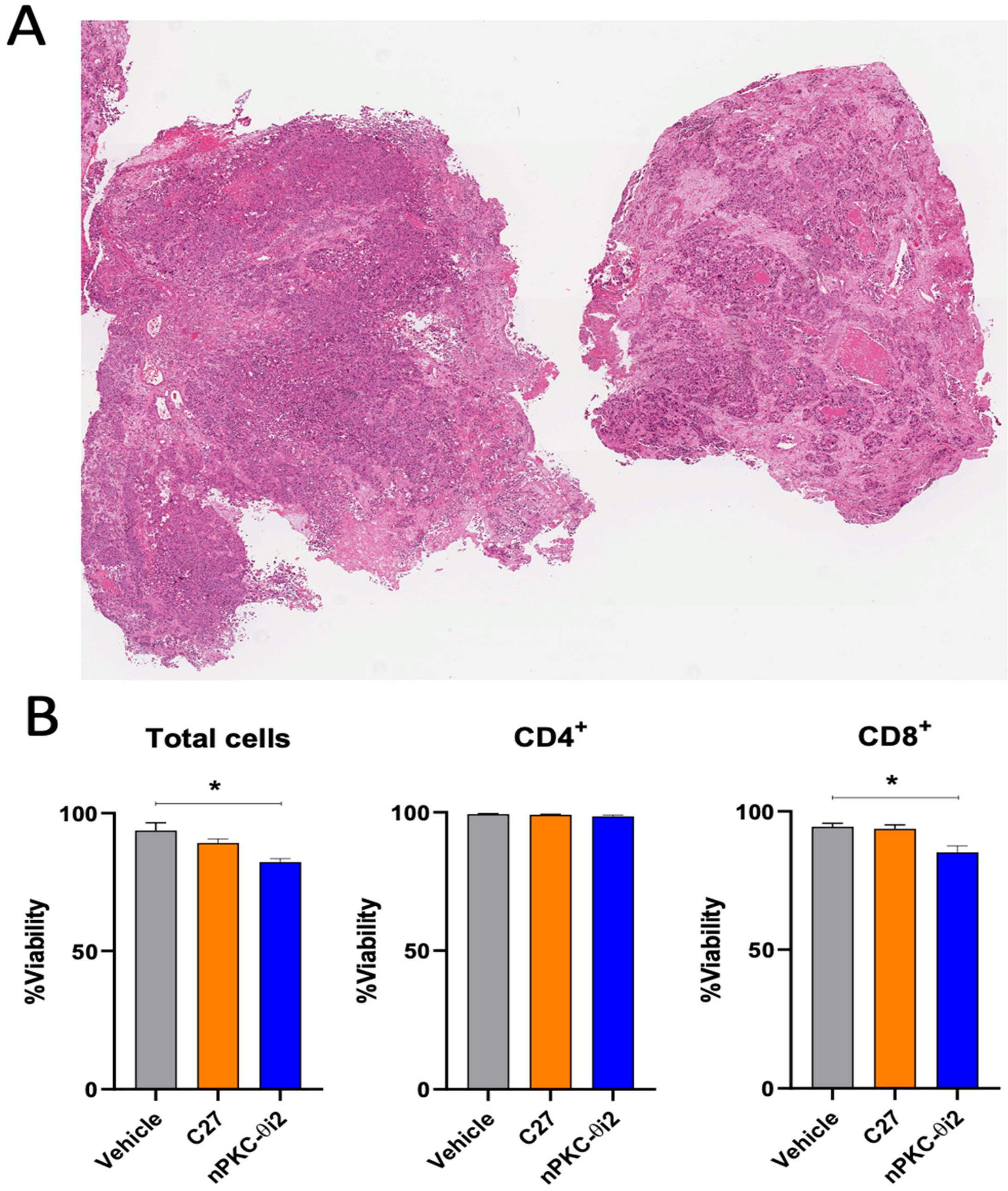

Figure S6. (**A**) Representative H&E-stained sections of a brain metastasis from a stage IV breast cancer patient matching the immunofluorescence staining in Figure 6F. (**B**) FACS plot showing % cell viability of total PBMCs, $CD4^+$ T cells, and $CD8^+$ T cells isolated from healthy donors and treated ex vivo with C27 or nPKC-θi2. Statistical significance is denoted * $p \leq 0.05$.
